# Icariin suppresses nephrotic syndrome by inhibiting pyroptosis and epithelial-to-mesenchymal transition

**DOI:** 10.1371/journal.pone.0298353

**Published:** 2024-07-12

**Authors:** Shuwen Duan, Zhaoran Ding, Can Liu, Xiaohui Wang, Enlai Dai

**Affiliations:** Department of Traditional Chinese and Western Medicine, Gansu University of Traditional Chinese Medicine, Lanzhou, China; National Institutes of Health, UNITED STATES OF AMERICA

## Abstract

**Context:**

Nephrotic syndrome(NS) has emerged as a worldwide public health problem. Renal fibrosis is the most common pathological change from NS to end-stage renal failure, seriously affecting the prognosis of renal disease. Although tremendous efforts have been made to treat NS, specific drug therapies to delay the progression of NS toward end-stage renal failure are limited. Epimedium is generally used to treat kidney disease in traditional Chinese medicine. Icariin is a principal active component of Epimedium.

**Methods:**

We used Sprague Dawley rats to establish NS models by injecting doxorubicin through the tail vein. Then icariin and prednisone were intragastric administration. Renal function was examined by an automatic biochemical analyzer. Pathology of the kidney was detected by Hematoxylin-Eosin and Masson staining respectively. Furthermore, RT-PCR, Enzyme-Linked Immunosorbent Assay, Immunohistochemistry, Western Blot and Terminal-deoxynucleotidyl Transferase Mediated Nick End Labeling staining were employed to detect the proteins related to pyroptosis and EMT. HK-2 cells exposed to doxorubicin were treated with icariin, and cell viability was assessed using the MTT. EMT was assessed using Enzyme-Linked Immunosorbent Assay and Western Blot.

**Results:**

The study showed that icariin significantly improved renal function and renal fibrosis in rats. In addition, icariin effectively decreased NOD-like receptor thermal protein domain associated protein 3,Caspase-1, Gasdermin D, Ly6C, and interleukin (IL)-1β. Notably, treatment with icariin also inhibited the levels of TGF-β, α-SMA and E-cadherin.

**Discussion and conclusions:**

It is confirmed that icariin can improve renal function and alleviate renal fibrosis by inhibiting pyroptosis and the mechanism may be related to epithelial-to-mesenchymal transition. Icariin treatment might be recommended as a new approach for NS.

## 1 Introduction

Nephrotic syndrome (NS) has emerged as a worldwide public health problem, which is characterized by edema, proteinuria, hypoproteinemia, and hyperlipidemia [1]. The accepted standard of care for patients with nephrotic syndrome is oral prednisone. Renal fibrosis is the most common pathological change from nephrotic syndrome to end-stage renal failure, which seriously affects the prognosis of renal disease [2]. Although tremendous efforts have been made to treat NS by preventing or retarding the progression of renal fibrosis, specific drug therapies to delay the progression of NS toward end-stage renal failure are limited [3].

Numerous studies have shown that pyroptosis is an important cause of renal disease progression [4–6]. Pyroptosis is a newly discovered lytic cell death [7]. Excessive pyroptosis forms an inflammatory microenvironment by triggering the release of inflammatory cytokines, such as IL-1β, IL-18 [8]. The inflammatory microenvironment is an inseparable factor of Epithelial-to-mesenchymal transition(EMT), an important mechanism of interstitial fibrosis [9–11]. Therefore, inhibition of pyroptosis may be a promising therapeutic strategy for renal fibrosis.

The accepted standard of care for patients with nephrotic syndrome is oral prednisone [12]. In recent years, with the increasing incidence of chronic kidney disease, the research and prevention of renal fibrosis have been paid more and more attention in the field of Traditional Chinese medicine (TCM). In TCM, Epimedium treats kidney disease. Icariin is a principal active component of Epimedium [13]. It has been demonstrated to have multiple pharmacological effects, including anti-fibrotic and anti-inflammatory [14, 15]. In addition, icariin is an inhibitor of NLRP3 inflammasome activation [16]. Therefore, we propose such an inference that icariin may have an intervening effect on renal fibrosis and this effect is related to the inhibition of NLRP3-mediated pyroptosis and EMT. This study intends to establish a rat model of NS by tail vein injection of doxorubicin, detect the expression of key molecules such as renal function, tubulointerstitial fibrosis, EMT, and pyroposis, and observe the changes after icariin intervention to confirm our inference.

## 2 Materials and methods

### 2.1 Chemicals

Doxorubicin(adriamycin) (HY-15142) was purchased from solarbio Biotechnology Co., Ltd (Beijing, China). Total cholesterol (2022011301), triglycerides (2021102601), creatinine (2021110302), urea nitrogen (2021111001) and albumin (2022011201) determination kits were purchased from Ruiyuan Biotechnology Co., Ltd (Ningbo, China).IL-1β ELISA kit were purchased from Feiya Biotechnology Co., Ltd(Nanjing, China).GSDMD polyclonal antibody(PA5-116815) was purchased from Invitrogen(USA), NLRP3 antibody(GTX00763), E-cadherin(GTX100443), α-SMA(GTX629702) from GeneTex(USA), caspase-1 antibody(YT5743) from immunoway(USA).

### 2.2 Animals and treatment protocols

Eight-week-old Specific Pathogen Free male rats (180-200g, certification No: SCXK 2019–0010) were supplied by Sibeifu Biotechnology Co., Ltd(Beijing, China). All rats were kept in an air-conditioned room at 23±2°C. For rat modeling, we refer to the scheme of Lee et al. and Yao et al [17, 18]. Thirty successfully prepared model rats were randomly divided into a doxorubicin group, an icariin group (50 mg/kg/d), and a prednisone group (6 mg/kg/d). On day 8 after tail vein injection of doxorubicin, rats were administered normal saline, icariin, or prednisone by gastric gavage once daily for 6 consecutive weeks.

### 2.3 Urine, blood and tissue

At the 2, 4, 6 and 8 weeks, the rats were placed in a metabolic cage for 24 h to collect urine samples. After general anesthesia, the rats were taken whole-blood samples through cardiac puncture. The blood was stored at -20°C for detection of albumin (ALB), total cholesterol (TC), triglycerides (TG), serum creatinine SCr), blood urea nitrogen (BUN) (automatic biochemical analyzer, No.82001, Hitachi diagnostic products Co., Ltd., Shanghai, China). The de-capsulated kidney was divided into two parts. One part of the renal tissues was fixed in 4% paraformaldehyde and embedded in paraffin. The remaining tissues were rapidly frozen by liquid nitrogen.

### 2.4 Histopathological examination

After dewaxing, Stain sections with Hematoxylin solution (3–5 min). Treat the section with Hematoxylin Differentiation solution, Hematoxylin Scott Tap Bluing, 85% ethanol for 5 min, 95% ethanol for 5 min, Stain sections with Eosin dye for 5 min. Dehydrate and observe with microscope inspection. The degree of renal fibrosis was assessed by Masson’s trichrome. Rinse the slices with 1% glacial acetic acid and then dehydration with two cup of anhydrous ethanol. Then, transparent sealing and observe with microscope(400×).

### 2.5 Immunohistochemistry

Kidney paraffin sections were deparaffinized in xylene and rehydrated. After blocking endogenous peroxidase activity, 3% BSA was added to the circle to evenly cover the tissue, and the tissues are sealed for 30 minutes at room temperature. The sealing solution is gently removed, the α-SAM(1:1000) and E-Cadherin(1:1000) prepared with PBS in a certain proportion is added to the sections, and the sections are placed flat in a wet box and incubated overnight at 4°C. After secondary antibody incubation, We carried out DAB chromogenic reaction and nucleus counterstaining. Visualize staining of tissue under a microscope((400×), acquisitive and analysis image.

### 2.6 Western blot

NLRP3, caspase-1, GSDMD, Ly6C and IL-1β in renal cortex were determined by Western blot. Take some undenatured protein solution and measured by the BCA protein. After SDS-PAGE, transferring the protein from the gel to the membrane. Put the transferred membrane into the TBST incubation tank for a quick Wash, Block the membrane by blocking buffer (25°C, 30 mins). Incubate the membrane with appropriate dilutions of primary antibody, overnight incubation at 4°C. Incubate the membrane with the recommended dilution of conjugated secondary antibody in blocking buffer (25°C, 30 mins). Wash the film three times with TBST, 5 minutes each time. Acquire image using darkroom development techniques for chemiluminescence, Perform ECL as described by the manufacturer, add ECL reagents for 1–2 mins at RT and capture western blot image using various durations of exposure.

### 2.7 Terminal-deoxynucleotidyl transferase mediated nick end labeling assay

After Deparaffinize and rehydrate, buffer is added to the tissues in the circle (10 min). Take appropriate amount of TDT enzyme, dUTP and buffer in the tunel kit according to the number of slices and tissue size and mix at 1:5:50 ratio. Add this mixture to objective tissue placed in a flat wet box (37°C, 2 h). DAPI counterstain in nucleus. Then incubate with DAPI solution (25°C, 30 mins), kept in dark place. Microscopic examination and collecting images through fluorescence microscope. Use ImageJ software to count the number of positive cells and total number of nucleated cells in the visual field, and take the number of positive cells/total number of nucleated cells as the percentage of TUNEL-positive cells.

### 2.8 Reverse transcription quantitative polymerase chain reaction(qRT-PCR)

Total RNA content was extracted using TRIzol kits and synthesized into complementary DNA with the help of reverse transcription kits. GAPDH serving as the endogenous control. Primers of PCR are shown in Table 1. Real-time fluorescence quantitative PCR was performed under the following conditions: pre-denaturation (95°C, 10 min), and 40 cycles of denaturation (95°C,15 s), annealing (60°C, 30 s) and extension (72°C, 30 s).

**Table 1:** qPCR primers.

genes	Forward Primer (5’-3’)	Reverse Primer (5’-3’)
NLRP3	AACGCCCTCGGTGACTTC	TGTTGCCTCGCAGGTAAAG
ASC	CCACCAACCCAAGCAAGA	GGTAGGACTGGGACTCCCTTA
caspase-1	TTTGAAGGACAAACCGAAGG	TGGAAGAGCAGAAAGCGATA
GSDMD	GGAGCTTCCACTTCTACGATG	GAGTCTGCCAGGTGTTAGGG

### 2.9 Cell culture and treatment

We cultured human kidney (HK)-2 cells in DMEM medium supplemented with 10% fetal bovine serum 100 units/ml of penicillin, 100μg/ml of streptomycin, and 1 mM of nonessential amino acids. We incubated the cells at 37°C in 5% CO_2_ and divided them into three treatment groups: control, doxorubicin (cells treated with 10μM of doxorubicin for 24 h) [19], doxorubicin + icariin (cells treated with icariin after 10μM Dox exposure).

### 2.10 Thiazolyl Blue Tetrazolium Bromide (MTT) assay

HK-2 cells were seeded in 96-well plates. After treatment with 10 μM of doxorubicin for 24 h, icariin at concentrations of 0, 1, 10, 20 and 50 μM for 24 h. 20μL MTT was added to the plates. After 4 h, 150 μL DMSO was added to each well to dissolve the formazan crystals. Absorbance was measured at 490 nm using a microplate reader.

### 2.11 Statistical analysis

All analysis was completed with SPSS version 26.0 Statistical Software. The experimental data were expressed as mean ± standard deviation. One-way analysis of variance was used to compare groups. The least significant difference (LSD) test was used when the variance was uniform, and tambane’s T2 test was used when the variance was not uniform. P<0.05 was considered to be statistically significant.

## 3 Results

### 3.1 Icariin improves body weight and renal function in the nephrotic syndrome rat induced by doxorubicin

**Fig 1:**
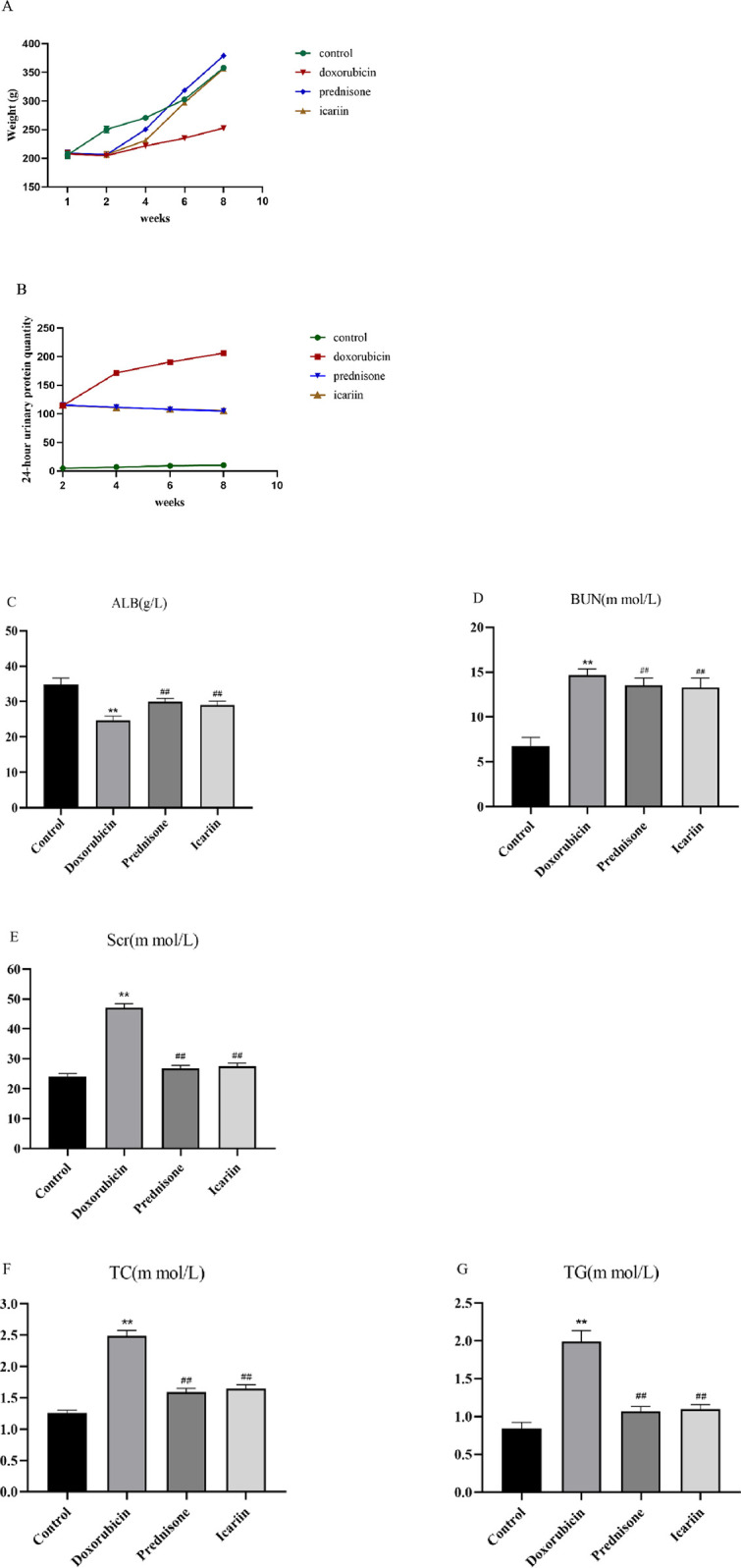
Icariin improves weight and renal function in the nephrotic syndrome rat induced by doxorubicin. (A) Weight of rats among different groups in different time.(B) 24-hour urinary protein quantity of rats among different groups in different time.(C-G) Biochemical parameters of rats among different groups. ** P < 0.01 versus control group; ## P < 0.01 versus doxorubicin group.

We weigh the rats regularly during the experiment (as shown in Table 2). Urine and blood of rats were collected for 24 h urinary protein and renal function tests (as shown in Tables 3 and 4). Data in Tables 2–4 are shown as Fig 1. We found that the weight and ALB decreased in the nephrotic syndrome rats induced by doxorubicin, while 24-hour urine protein, BUN, Scr, TC, and TG increased. These tests revealed the renal function was damaged in the doxorubicin rats. Compared with the doxorubicin group, the weight, 24-hour urine protein, BUN, SCR, TC, and TG of the prednisone group and the icariin group decreased, while ALB increased. And there was no significant differences between the icariin group and the prednisone group. This suggests that prednisone and icariin can improve kidney function and have similar effects.

**Table 2:** Weight of rats among different groups in different time.

Groups (n = 10)	0 week(g)	2weeks(g)	treatment		
			4weeks(g)	6weeks(g)	8weeks(g)
Control	206±5.85	250±5.77	271±4.10	303±4.50	358±4.56
Doxorubicin	208±6.06	204±4.40**	220±4.74**	222±4.23**	235±4.71**
Prednisone	209±5.41	206±3.71	249.90±2.85^##^	319±4.80^##^	379±3.98^##^
Icariin	209±5.59	206±4.81	231±3.02^##^	297±3.02^##^	356±3.84^##^

*P < 0.05

**P < 0.01 versus control group

^#^ P < 0.05

^##^ P < 0.01 versus doxorubicin group.

**Table 3:** 24-hour urinary protein quantity of rats among different groups in different time.

Groups (n = 10)	2weeks (mg/24h)	treatment		
		4weeks (mg/24h)	6weeks (mg/24h)	8weeks (mg/24h)
Control	4.83±0.88	6.67±0.91	9.03±1.13	10.34±0.91
Doxorubicin	114.25±1.16**	171.52±4.20**	190.60±2.84**	206.24±2.48**
Prednisone	115.60±2.81	111.47±3.66^##^	107.69±2.23^##^	104.93±2.40^##^
Icariin	114.84±1.46	110.64±2.72^##^	108.03±3.20^##^	105.26±3.05^##^

*P < 0.05

**P < 0.01 versus control group

^#^ P < 0.05

^##^ P < 0.01 versus doxorubicin group.

**Table 4:** Biochemical parameters of rats among different groups.

Groups (n = 10)	ALB(g/L)	BUN(m mol/L)	Scr(m mol/L)	TC(m mol/L)	TG(m mol/L)
Control	34.88±1.75	6.75±0.98	23.96±1.08	1.25±0.47	0.84±0.82
Doxorubicin	24.74±1.16**	14.67±0.68**	47.09±1.40**	2.49±0.88**	1.99±0.15**
Prednisone	29.37±1.09^##^	13.50±0.83^##^	38.29±0.99^##^	1.59±0.58^##^	1.07±0.06^##^
Icariin	28.11±2.41^##^	13.28±1.05^##^	38.65±0.71^##^	1.64±0.64^##^	1.10±0.06^##^

*P < 0.05

**P < 0.01 versus control group

^#^P < 0.05

^##^P < 0.01 versus doxorubicin group.

### 3.2 Icariin alleviates fibrosis in the nephrotic syndrome rat induced by doxorubicin

**Fig 2:**
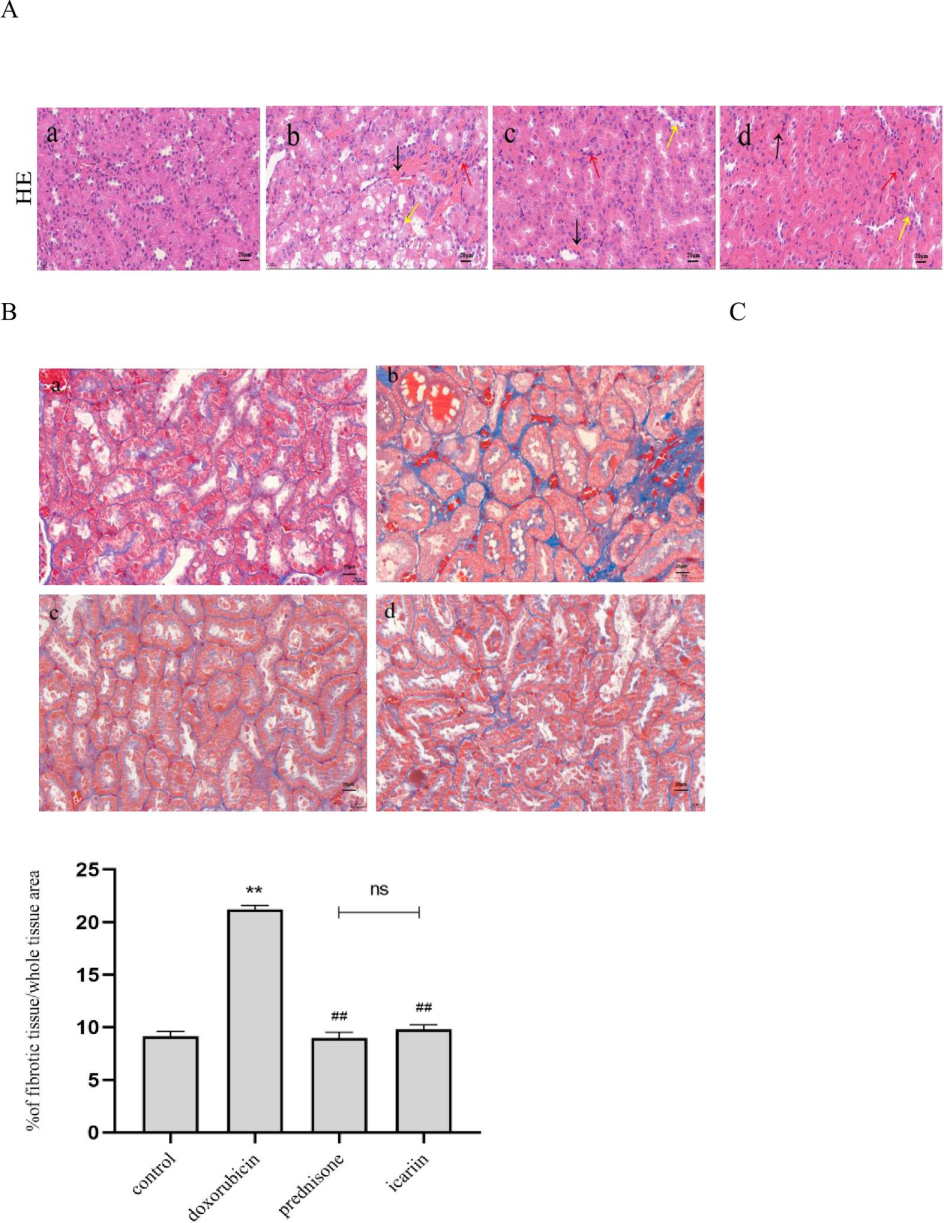
Icariin suppresses tubulointerstitial fibrosis in the kidney tissues of nephrotic syndrome rats. (A) Representative photomicrographs of hematoxylin and eosin-stained kidney sections of rats from different groups: (a) control group, (b) doxorubicin group, (c) prednisone group, (d) icariin group. Scale bar: 20 μm. The red arrows stand for the invasion of inflammatory cells, the black arrows stand for fibrosis, the yellow arrows stand for tubular atrophy. (B) Representative photomicrographs of kidney sections by Masson’s trichrome staining in each group of rats. Scale bar: 20 μm. (C) The degree of tubulointerstitial collagen deposition were assessed.** P < 0.01 versus control group; ^##^ P < 0.01 versus doxorubicin group.

Kidney tissues were collected for Hematoxylin-Eosin (H&E) and Masson staining to evaluate renal morphological structure damages(as shown in Fig 2). The results of H&E staining showed that renal tubular epithelial cells were arranged neatly with clear intercellular spaces in the control group. There is no dilation of renal tubules or infiltration of inflammatory cells. In the doxorubicin group, tubular epithelial cells showed irregular arrangement. Renal tubules were dilated, and the area of the renal tubulointerstitium was enlarged. A large number of inflammatory cells infiltrated into the renal tubulointerstitium. In the prednisone and icariin group, the pathological morphology of renal tissue was improved. The dilation of renal tubules, the infiltration of inflammatory cells in the renal interstitium, the proliferation of fibers were reduced compared with the doxorubicin group. And there was no significant differences between the icariin group and the prednisone group. It suggests that icariin can reduce renal morphological structure damages.

Masson staining showed a small amount of blue collagen fiber deposition in the kidney tissue of rats in the control group, and a large amount of blue-gray collagen fiber deposition in the kidney tissue of rats in the doxorubicin group. Compared with the control group, the relative area of renal interstitial fibrosis in the doxorubicin group increased (P<0.01). Compared with the doxorubicin group, the relative area of renal interstitial fibrosis in the prednisone group and icariin group decreased (P<0.01), and there was no statistical differences between the prednisone group and the icariin group. It indicates that icariin can reduce fibrosis in rats with nephrotic syndrome.

### 3.3 Icariin alleviates the levels of inflammatory factors in the nephrotic syndrome rat induced by doxorubicin

**Fig 3:**
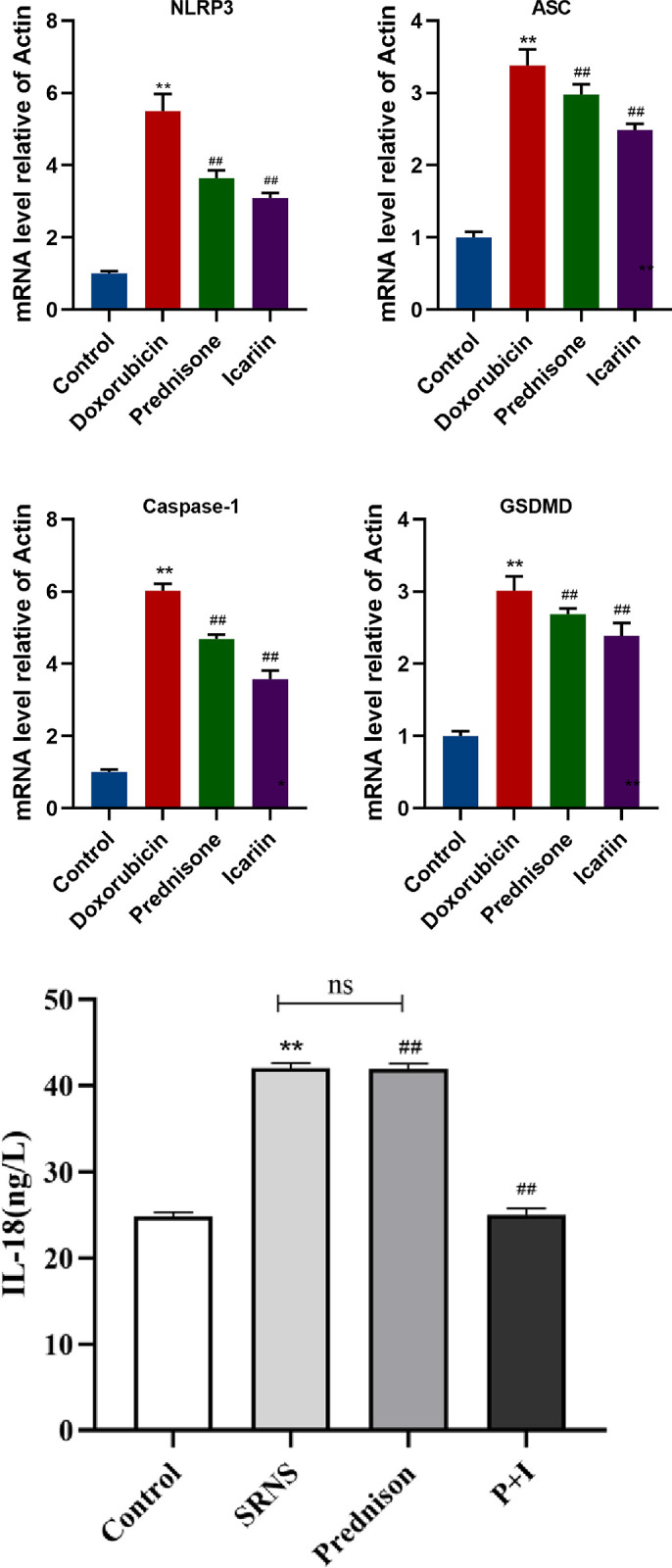
Icariin alleviates the levels of inflammatory cells in the nephrotic syndrome rat induced by doxorubicin. The NLRP3, ASC, caspase-1, GSDMD were detected by qRT-PCR. IL-18 were detected by ELISA. ** P < 0.01 versus control group; ^##^ P < 0.01 versus doxorubicin group.

The NLRP3, ASC, caspase-1, GSDMD were detected by qRT-PCR. The TDF-β and IL-18 were detected by ELISA. Compared with the control group, NLRP3, ASC, caspase-1, GSDMD and IL-18 in the doxorubicin group showed an upward trend. Compared with the doxorubicin group, NLRP3, caspase-1, GSDMD and IL-18 in the icariin group and prednisone group showed a downward trend (as shown in Fig 3). It implies that icariin can reduce the levels of some major inflammatory factors associated with pyroptosis.

### 3.4 Icariin alleviates pyroptosis in rats with doxorubicin induced nephrotic syndrome

**Fig 4:**
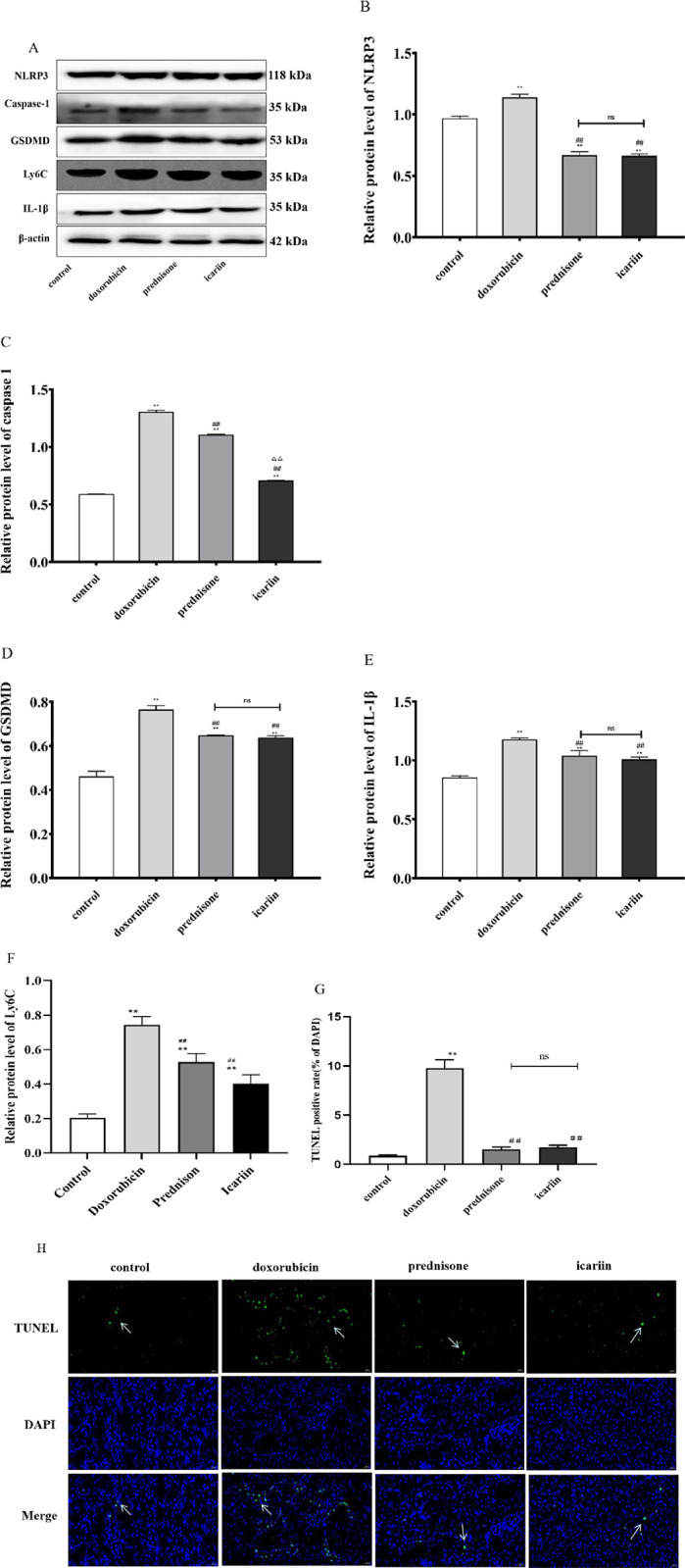
Icariin alleviates pyroptosis in nephrotic syndrome rats. (A) Kidney cortex tissues were collected and analyzed by Western blotting to detect NLRP3, caspase-1, GSDMD, Ly6C and IL-1β, respectively. The β-actin protein levels served as internal controls. (B-F) Data are expressed as the ratio of NLRP3,caspase-1, GSDMD, Ly6C and IL-1β over β-actin and are given as mean 1 standard deviation of at least three independent determinations. (G)TUNEL staining was used to evaluate the cell death. Nucleus is blue, positive cells are green. scale bar: 20 μm. (H)Statistical analysis of the percentage of TUNEL-positive cells normalised by the control group. ** P < 0.01 versus control group; ^##^ P < 0.01 versus doxorubicin group.

The NLRP3, caspase-1, GSDMD, Ly6C and IL-1β in renal tissue were detected by Western blot. Compared with the control group, NLRP3, caspase-1, GSDMD, Ly6C and IL-1β in the doxorubicin group showed an upward trend. Compared with the doxorubicin group, NLRP3, caspase-1, GSDMD, Ly6C and IL-1β in the icariin group and prednisone group showed a downward trend. There was no significant differences between the icariin group and the prednisone group. TUNEL assay showed that only a few TUNEL-positive cells were found in the control group. Compared with the control group, the percentage of TUNEL-positive cells in the doxorubicin group was significantly increased; Compared with the doxorubicin group, the percentage of TUNEL-positive cells in the prednisone group and the icariin group decreased significantly. Compared with the prednisone group, the percentage of TUNEL-positive cells in the icariin group decreased without a statistical difference (as shown in Fig 4). The results of western blot and TUNEL assay suppose that doxorubicin-induced NS is related to pyroptosis, and icariin alleviates pyroptosis.

### 3.4 Icariin alleviates EMT in rats with doxorubicin-induced nephrotic syndrome

**Fig 5:**
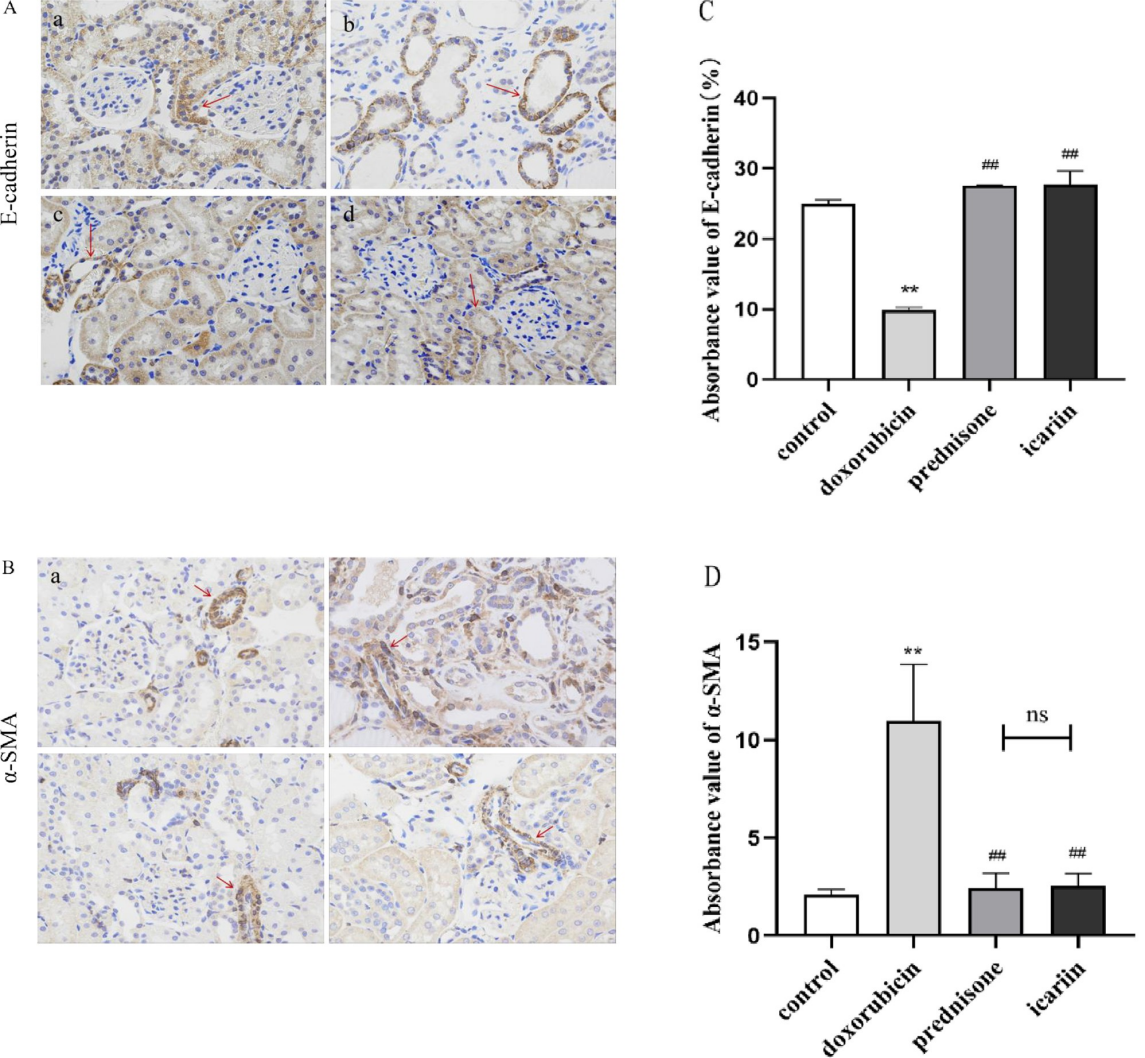
Icariin inhibited the EMT process in the kidney tissues of nephrotic syndrome rats. (A, B)The location and expression of E-cadherin and α-SMA were determined by immunohistochemical staining in the kidney sections of rats from different groups: (a) control group, (b) doxorubicin group, (c) prednisone group, (d) icariin group. Scale bar:20 μm. The red arrows stand for positive cells. (C, D) Semi-quantitative immunohistochemical analysis of the EMT-related protein expression in different groups. ** P < 0.01 versus control group; ^##^ P < 0.01 versus doxorubicin group.

To assess the effect of icariin on the EMT in rats with doxorubicin-induced NS, we analyzed the expression of several key proteins of EMT by immunohistochemistry. Compared with the control group, α-SMA showed an upward trend, while E-cadherin showed a downward trend in the doxorubicin group. Compared with the doxorubicin group, α-SMA showed a downward trend, and E-cadherin showed an upward trend in the icariin group and prednisone group. Compared to the icariin group with the prednisone group, there is no statistical difference in the quantitative analysis of α-SMA protein and E-cadherin protein. The results suppose that icariin alleviates EMT in rats with doxorubicin- induced NS. (as shown in Fig 5)

### 3.5 Icariin inhibits EMT in HK-2 cells

**Fig 6:**
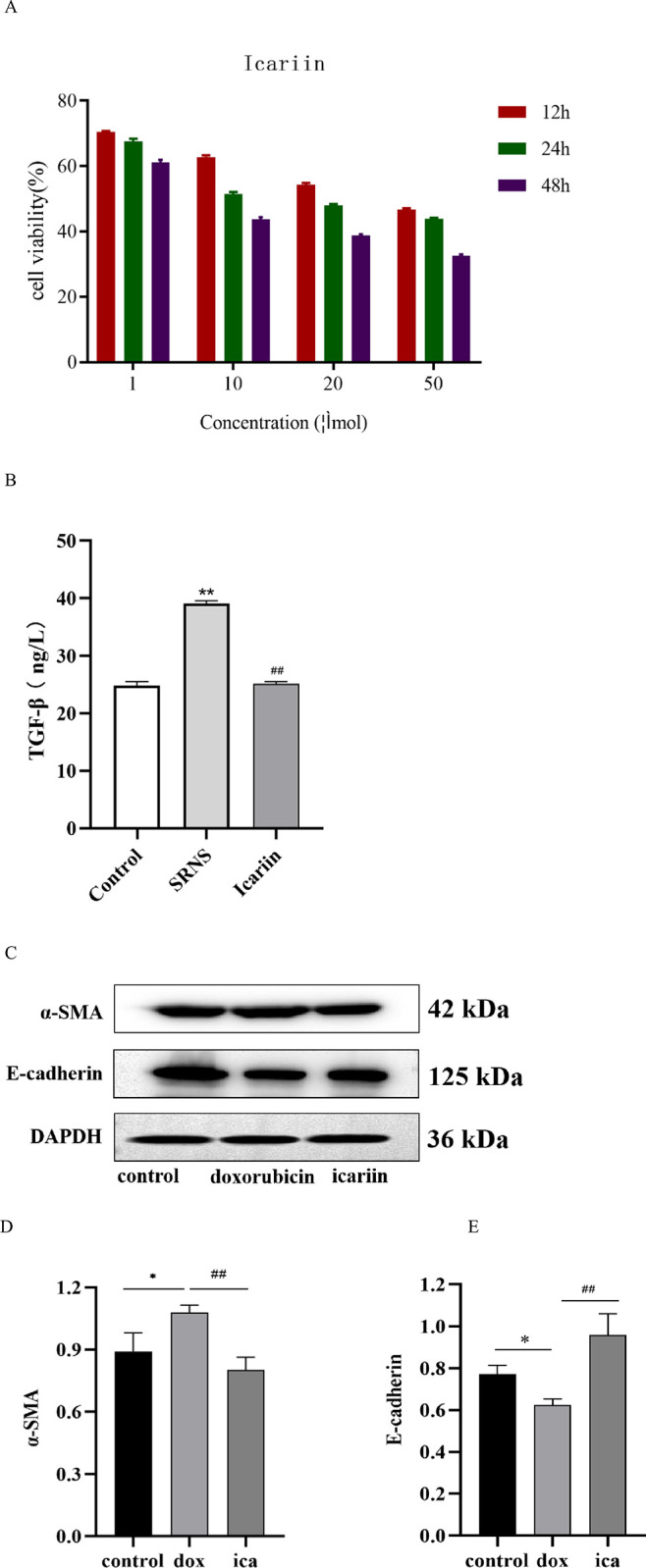
Icariin inhibits EMT in HK-2 cells. (A) MTT assy shows that the optimal dosage of icariin is 10 μM for 24 hours. (B) ELISA implies that the level of TGF-β can be suppressed by icariin. (C-E) WB suggests that α-SMA protein can be suppressed by icariin and E-cadherin protein can be stimulated.

MTT assy shows that the optimal dosage of icariin is 10 μM for 24 hours (as shown in Fig 6A). ELISA implies that the level of TGF-β can be suppressed by icariin (as shown in Fig 6B). WB suggests that α-SMA protein and E-cadherin protein can be suppressed by icariin (as shown in Fig 6C). These results provide evidence that icariin inhibits EMT in HK-2 cells.

## 4 Discussion

NS refers to hyperproteinuria with associated hypoproteinemia, edema, and hyperlipidemia [20]. Renal fibrosis is one of the most important pathological manifestations in the process of nephrotic syndrome. Renal fibrosis refers to the pathological manifestation of ECM accumulation caused by the proliferation of interstitial fibroblasts, an increase of interstitial protein synthesis, and inhibition of matrix degradation under the action of various pathogenic factors [21]. Pyroptosis is a new way of cell death discovered in recent years. Many studies have shown that pyroptosis is a new frontier in kidney diseases [22–24]. The important factors related to pyroptosis are NLRP3, caspase-1, GSDMD, IL-18 and IL-1β. NLRP3 is the key protein in pyroptosis, and knocking out NLRP3 can inhibit pyroptosis. NLRP3 inflammasomes are constituted by a sensor molecule, the adap-tor apoptosis-associated speck-like protein containing a CARD (ASC) and pro-caspase-1 [25]. Caspase-1 (originally named ICE for the interleukin-converting enzyme), belonging to the inflammatory caspase group, was the first caspase identified via its activity of processing pro-IL-1β and pro-IL-18 into mature IL-1β and IL-18 [26]. It was recently shown that cleavage of GSDMD by the inflammatory caspases-1 is critical for pyroptosis to occur [27]. The released N-terminus of GSDMD then binds to phosphoinositide in the plasma membrane to form membrane pores, resulting in cell swelling and eventual lysis, accompanied by IL-1β and IL-18 released from the cell [28].Excessive pyroptosis triggers intense inflammation by releasing inflammatory cytokines and dangerous molecules. Inflammatory microenvironment can promote the onset of EMT, an important mechanism of renal fibrosis [29, 30]. EMT is a process in which epithelial cells change their phenotype and eventually transform into myofibroblasts, which is one of the main reasons for ECM aggregation. Therefore, we speculate that the effect of pyroptosis on renal fibrosis may be related to EMT. The sign of EMT is the decrease of E-cadherin and the increase of α-SMA. Normally, the epithelial cells of renal tubules are connected by E-cadherin to maintain the function of renal tubules [31]. α-SMA is a kind of actin and a molecular marker of MF, which is the main effector cell of extracellular matrix deposition in the process of renal fibrosis. When EMT occurs, tight junctions between renal tubular epithelial cells disappear, accompanied by a decrease in E-cadherin and an increase in α-SMA.

This study showed that icariin had an improved effect on pathological changes and functions of the kidneys of rats. This is consistent with the research of Jia Z, Zhang L, Chen HA et al [32–34]. Its mechanism of action is the focus of this thesis. The expression of NLRP3, Casapase-1, GSDMD, IL-1β, and α-SMA in the renal tissue of nephrotic syndrome rats increased, E-cadherin decreased, and Tunel staining showed that an increase of renal tubular epithelial cells with broken nuclei. TUNEL is an acronym for terminal deoxynucleotidyl transferase biotin-dUTP nick end labeling. Pyroptosis can be proven based on joint TUNEL-WB evaluation. The results indicate that pyroptosis and EMT occur. After intervention by icariin, the damage of renal tubulointerstitium in rats was significantly reduced, and the expression of NLRP3, Caspase-1, GSDMD, and IL-1β protein in renal tissue was reduced, renal tubular epithelial cells with broken nuclei decreased. The results indicate that icariin can reduce inflammatory reactions by inhibiting pyroptosis. Our further research found that the expression of α-SMA in the kidney tissue of rats with NS increased, and the expression of E-cadherin decreased, indicating that EMT occurred. After icariin treatment, the expression of α-SMA in rat kidney tissue decreased. In contrast, the expression of E-cadherin increased, indicating that icariin also had an inhibitory effect on EMT in kidney tissue. Through this study, we found that icariin plays a role in NS by inhibiting pyroptosis and EMT.

## 5. Conclusion

In summary, our results showed that the therapeutic potential of icariin as a countermeasure for inhibiting renal tubular epithelial cells’ pyroptosis and EMT. In addition, no mortality was observed in the rats treated only with icariin in the experiment. However, some limitations of this study should be noted. The safety of icariin for the treatment of NS needs thoroughly investigate. We did not evaluate the changes of EMT in renal tubular epithelial cells after NLRP3 knockout, and more research is still needed.

## Supporting information

S1 Dataset (DOC)

S1 Raw images (DOC)

S2 Raw images (DOC)
