# Supplementary material for: Icariin suppresses nephrotic syndrome by inhibiting pyroptosis and epithelial-to-mesenchymal transition
Source: PLoS One. 2024 Jul 12;19(7):e0298353. doi: 10.1371/journal.pone.0298353 (PMC11244770; doi:10.1371/journal.pone.0298353)
Supplement: S1 Dataset — (DOC) [file pone.0298353.s001.doc]

**Weight of rats among different groups in different time.**

|  | control |  |  |  |  |  |  |  |  |  |
| --- | --- | --- | --- | --- | --- | --- | --- | --- | --- | --- |
| 0week | 209 | 201 | 198 | 205 | 206 | 211 | 214 | 210 | 208 | 196 |
| 2week | 254 | 250 | 241 | 258 | 245 | 245 | 257 | 248 | 256 | 249 |
| 4week | 274 | 265 | 269 | 278 | 273 | 269 | 271 | 270 | 265 | 274 |
| 6week | 305 | 307 | 295 | 303 | 299 | 306 | 301 | 306 | 308 | 297 |
| 8week | 359 | 354 | 362 | 355 | 361 | 357 | 359 | 367 | 356 | 351 |
|  | doxorubicin |  |  |  |  |  |  |  |  |  |
| 0week | 212 | 196 | 213 | 209 | 213 | 206 | 208 | 209 | 198 | 212 |
| 2week | 206 | 212 | 206 | 198 | 204 | 203 | 205 | 205 | 197 | 208 |
| 4week | 225 | 223 | 229 | 215 | 220 | 218 | 217 | 223 | 219 | 226 |
| 6week | 231 | 230 | 238 | 227 | 239 | 240 | 238 | 237 | 231 | 239 |
| 8week | 250 | 253 | 261 | 254 | 257 | 254 | 255 | 246 | 245 | 253 |
|  | prednisone |  |  |  |  |  |  |  |  |  |
| 0week | 211 | 213 | 211 | 214 | 196 | 209 | 205 | 214 | 207 | 209 |
| 2week | 207 | 209 | 209 | 211 | 204 | 208 | 205 | 208 | 198 | 204 |
| 4week | 246 | 254 | 252 | 246 | 247 | 250 | 251 | 249 | 253 | 251 |
| 6week | 324 | 318 | 324 | 314 | 320 | 316 | 327 | 319 | 316 | 312 |
| 8week | 383 | 386 | 373 | 384 | 376 | 379 | 377 | 380 | 379 | 377 |
|  | icariin |  |  |  |  |  |  |  |  |  |
| 0week | 211 | 198 | 208 | 203 | 215 | 207 | 215 | 206 | 214 | 212 |
| 2week | 207 | 206 | 212 | 201 | 213 | 201 | 209 | 200 | 211 | 204 |
| 4week | 229 | 231 | 228 | 238 | 230 | 231 | 234 | 231 | 230 | 228 |
| 6week | 299 | 301 | 297 | 293 | 299 | 292 | 295 | 298 | 299 | 300 |
| 8week | 347 | 355 | 354 | 353 | 358 | 357 | 356 | 355 | 361 | 359 |

1. **hour urinary protein quantity of rats among different groups in different time.**

|  | 2week | 4week | 6week | 8week |
| --- | --- | --- | --- | --- |
| control | 4.82 | 6.72 | 8.58 | 9.56 |
|  | 5.32 | 6.66 | 9.66 | 10.84 |
|  | 4.95 | 7.39 | 10.69 | 11.96 |
|  | 4.01 | 6.35 | 8.95 | 9.37 |
|  | 3.79 | 5.64 | 7.92 | 8.99 |
|  | 4.82 | 6.93 | 8.05 | 10.08 |
|  | 6.85 | 8.86 | 10.49 | 11.36 |
|  | 4.91 | 5.88 | 7.22 | 10.53 |
|  | 3.95 | 5.98 | 8.98 | 10.27 |
|  | 4.91 | 6.26 | 9.78 | 10.44 |
| doxorubicin | 112.79 | 166.63 | 190.65 | 205.34 |
|  | 113.53 | 162.37 | 189.37 | 201.39 |
|  | 115.48 | 169.73 | 187.83 | 205.38 |
|  | 113.97 | 172.66 | 193.53 | 204.43 |
|  | 114.58 | 175.58 | 194.19 | 208.87 |
|  | 115.43 | 173.59 | 194.93 | 208.38 |
|  | 112.69 | 171.26 | 191.32 | 209.87 |
|  | 114.93 | 174.47 | 189.49 | 207.47 |
|  | 115.82 | 174.91 | 187.94 | 205.28 |
|  | 113.28 | 173.95 | 186.79 | 205.97 |
| prednisone | 116.48 | 103.28 | 109.68 | 106.64 |
|  | 114.48 | 112.36 | 107.93 | 103.84 |
|  | 119.46 | 113.28 | 105.25 | 104.57 |
|  | 117.27 | 114.39 | 106.97 | 103.35 |
|  | 115.34 | 112.19 | 110.69 | 101.85 |
|  | 113.74 | 110.16 | 109.38 | 109.58 |
|  | 119.65 | 115.73 | 107.28 | 105.36 |
|  | 113.35 | 110.29 | 103.08 | 107.59 |
|  | 110.47 | 108.39 | 108.64 | 103.99 |
|  | 115.74 | 114.58 | 107.96 | 102.48 |
| icariin | 115.53 | 109.47 | 108.76 | 109.57 |
|  | 116.59 | 112.45 | 108.99 | 105.82 |
|  | 114.98 | 112.53 | 101.11 | 107.86 |
|  | 114.73 | 109.39 | 103.97 | 103.44 |
|  | 112.09 | 104.35 | 111.46 | 102.27 |
|  | 115.57 | 112.68 | 110.38 | 110.27 |
|  | 114.49 | 110.63 | 107.82 | 103.19 |
|  | 116.95 | 113.95 | 110.38 | 104.75 |
|  | 114.24 | 109.47 | 107.62 | 103.94 |
|  | 113.22 | 111.47 | 109.85 | 101.44 |

**Biochemical parameters of rats among different groups.**

|  | control | doxorubicin | prednisone | icariin |
| --- | --- | --- | --- | --- |
| ALB | 35.65 | 24.35 | 30.84 | 29.97 |
|  | 37.54 | 26.45 | 29.64 | 30.53 |
|  | 36.42 | 25.75 | 28.45 | 27.89 |
|  | 32.57 | 23.65 | 28.85 | 28.48 |
|  | 33.64 | 23.68 | 29.68 | 28.09 |
|  | 35.45 | 24.64 | 28.98 | 27.48 |
|  | 34.87 | 25.57 | 30.85 | 29.46 |
|  | 33.36 | 24.79 | 30.41 | 30.66 |
|  | 32.68 | 22.75 | 31.01 | 28.57 |
|  | 36.64 | 25.75 | 30.97 | 28.99 |
| BUN | 6.84 | 15.24 | 14.48 | 12.39 |
|  | 5.97 | 14.35 | 13.65 | 14.48 |
|  | 8.04 | 14.98 | 12.98 | 13.56 |
|  | 7.93 | 15.25 | 14.35 | 15.03 |
|  | 5.39 | 13.99 | 12.59 | 12.19 |
|  | 5.93 | 14.47 | 13.47 | 14.08 |
|  | 7.38 | 15.18 | 14.11 | 13.33 |
|  | 6.24 | 13.67 | 12.01 | 11.97 |
|  | 5.95 | 13.93 | 13.12 | 12.32 |
|  | 7.79 | 15.67 | 14.28 | 13.48 |
| Scr | 23.83 | 47.38 | 26.39 | 27.12 |
|  | 24.46 | 46.94 | 27.45 | 28.19 |
|  | 25.64 | 48.59 | 28.32 | 28.56 |
|  | 23.57 | 46.64 | 27.63 | 25.57 |
|  | 22.27 | 47.57 | 26.64 | 28.75 |
|  | 25.01 | 48.03 | 27.94 | 27.85 |
|  | 23.65 | 46.86 | 25.79 | 26.78 |
|  | 24.96 | 48.99 | 26.18 | 28.37 |
|  | 22.54 | 45.57 | 25.39 | 26.75 |
|  | 23.66 | 44.28 | 26.05 | 26.16 |
| TC | 1.25 | 2.46 | 1.59 | 1.65 |
|  | 1.19 | 2.36 | 1.54 | 1.59 |
|  | 1.28 | 2.55 | 1.62 | 1.72 |
|  | 1.25 | 2.38 | 1.46 | 1.55 |
|  | 1.33 | 2.49 | 1.56 | 1.73 |
|  | 1.26 | 2.39 | 1.62 | 1.62 |
|  | 1.32 | 2.56 | 1.64 | 1.68 |
|  | 1.20 | 2.48 | 1.62 | 1.58 |
|  | 1.26 | 2.57 | 1.59 | 1.61 |
|  | 1.21 | 2.61 | 1.66 | 1.71 |
| TG | 0.85 | 2.01 | 1.02 | 1.12 |
|  | 0.76 | 1.98 | 1.11 | 1.04 |
|  | 0.89 | 2.36 | 1.16 | 1.13 |
|  | 0.92 | 1.89 | 1.13 | 1.07 |
|  | 0.72 | 2.08 | 1.09 | 1.17 |
|  | 0.94 | 1.99 | 1.04 | 1.15 |
|  | 0.89 | 1.86 | 1.12 | 1.07 |
|  | 0.76 | 1.91 | 1.05 | 0.97 |
|  | 0.92 | 1.89 | 0.97 | 1.15 |
|  | 0.77 | 1.95 | 0.99 | 1.10 |

**ELISA**

IL-18

| Control | Doxorubicin | Prednison | Icariin |
| --- | --- | --- | --- |
| 24.9753 | 41.7919 | 42.3901 | 25.877 |
| 24.0258 | 42.1768 | 41.3731 | 24.1508 |
| 24.406 | 41.2877 | 41.1435 | 24.3543 |
| 25.0461 | 41.2582 | 42.5305 | 25.0294 |
| 25.4348 | 42.8794 | 42.0737 | 25.9116 |
| 24.866 | 42.2834 | 41.6597 | 25.4007 |
| 25.287 | 42.4469 | 42.5673 | 24.6361 |

TGF-β

| Control | Doxorubicin | Prednison | Icariin |
| --- | --- | --- | --- |
| 24.5478 | 38.6991 | 38.4066 | 24.9164 |
| 24.1352 | 38.5567 | 37.26 | 25.9799 |
| 24.2062 | 39.3537 | 38.5358 | 25.0416 |
| 24.2486 | 38.6812 | 38.9753 | 24.7399 |
| 25.772 | 39.217 | 38.1855 | 25.4957 |
| 25.6866 | 38.9771 | 37.8029 | 24.7443 |
| 25.4123 | 39.2761 | 37.6446 | 25.0155 |

**PCR**

NLRP3

| Control | Doxorubicin | Prednison | Icariin |
| --- | --- | --- | --- |
| 1.060140093 | 5.997057989 | 3.875157433 | 3.125863809 |
| 1.016954352 | 5.07798074 | 3.590674616 | 3.213743803 |
| 0.922905555 | 5.442445 | 3.468363057 | 2.95724736 |

ASC

| Control | Doxorubicin | Prednison | Icariin |
| --- | --- | --- | --- |
| 1.071929109 | 3.630603794 | 3.138792152 | 2.445635668 |
| 1.014106731 | 3.294842488 | 2.888277776 | 2.428742428 |
| 0.91396416 | 3.204744847 | 2.908367337 | 2.585081021 |

Caspase-1

| Control | Doxorubicin | Prednison | Icariin |
| --- | --- | --- | --- |
| 1.062397679 | 6.221764979 | 4.78103809 | 3.42789733 |
| 1.019119974 | 6.009828823 | 4.715216126 | 3.856584976 |
| 0.918482348 | 5.845489738 | 4.554598555 | 3.451740242 |

GSDMD

| Control | Doxorubicin | Prednison | Icariin |
| --- | --- | --- | --- |
| 1.038478149 | 3.236581561 | 2.778817061 | 2.304527303 |
| 1.038478149 | 2.937259765 | 2.628921129 | 2.592727995 |
| 0.923043702 | 2.856940242 | 2.647206722 | 2.257100817 |
