# Supplementary figures and images for: Icariin suppresses nephrotic syndrome by inhibiting pyroptosis and epithelial-to-mesenchymal transition
Source: PLoS One. 2024 Jul 12;19(7):e0298353. doi: 10.1371/journal.pone.0298353 (PMC11244770; doi:10.1371/journal.pone.0298353)

NLRP3


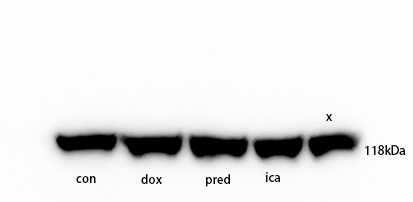


Caspase-1


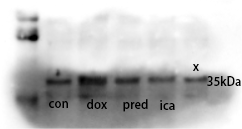


GSDMD


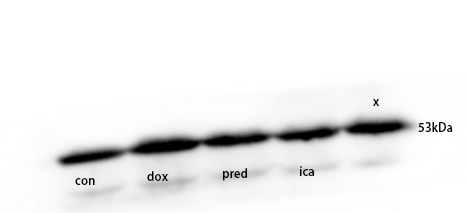


IL-1β


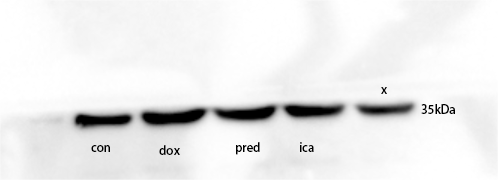


Ly6C


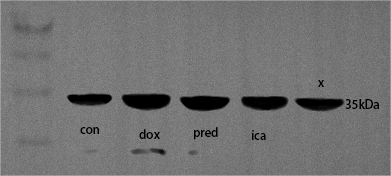


α-SMA


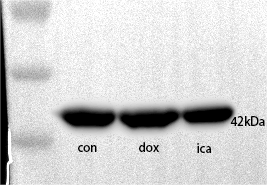


1. Cadherin


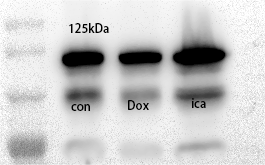

Supplement: S1 Raw images — (DOC) [file pone.0298353.s002.doc]
