# Supplementary material for: Icariin suppresses nephrotic syndrome by inhibiting pyroptosis and epithelial-to-mesenchymal transition
Source: PLoS One. 2024 Jul 12;19(7):e0298353. doi: 10.1371/journal.pone.0298353 (PMC11244770; doi:10.1371/journal.pone.0298353)

**HE**

Control


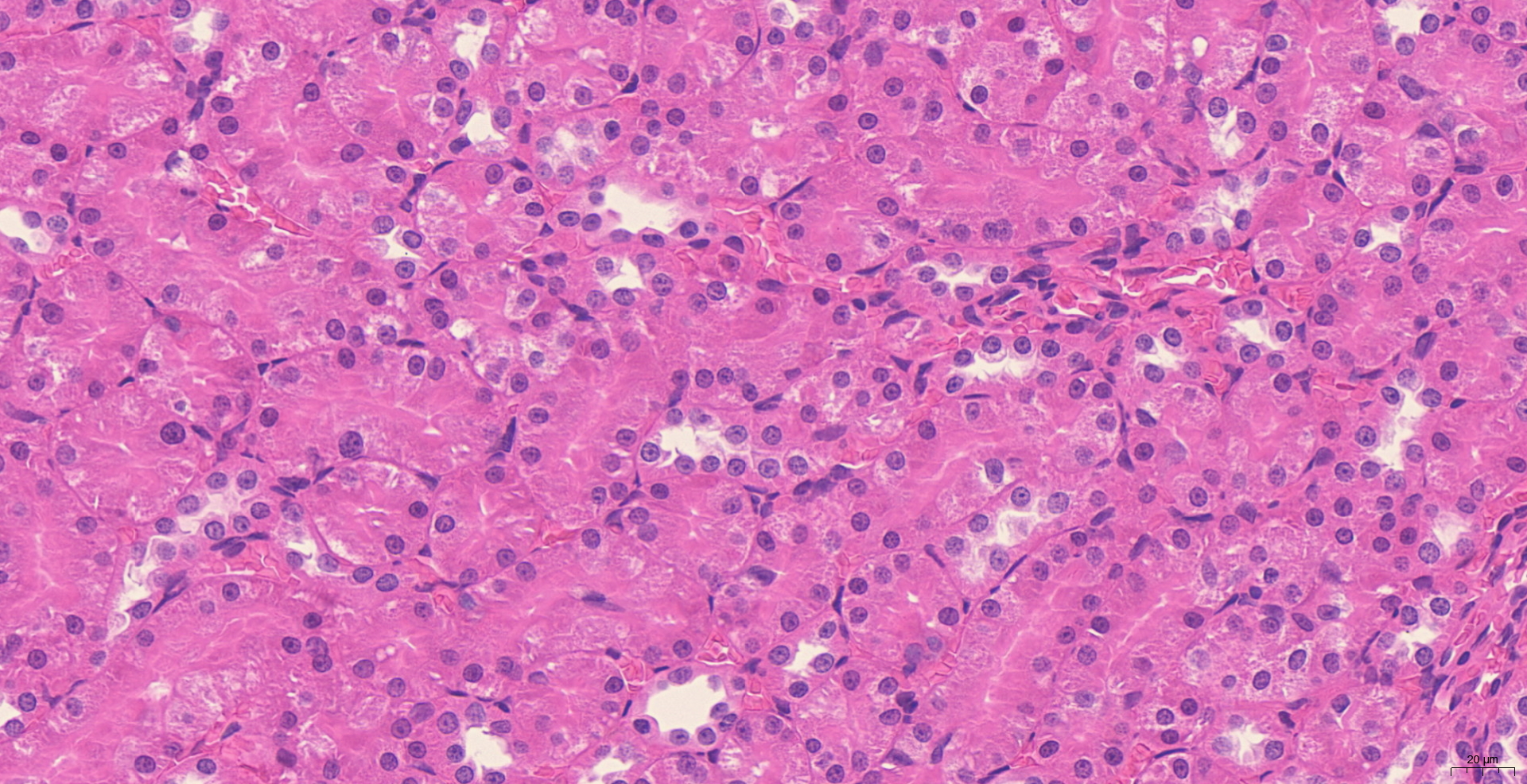


Doxorubicin


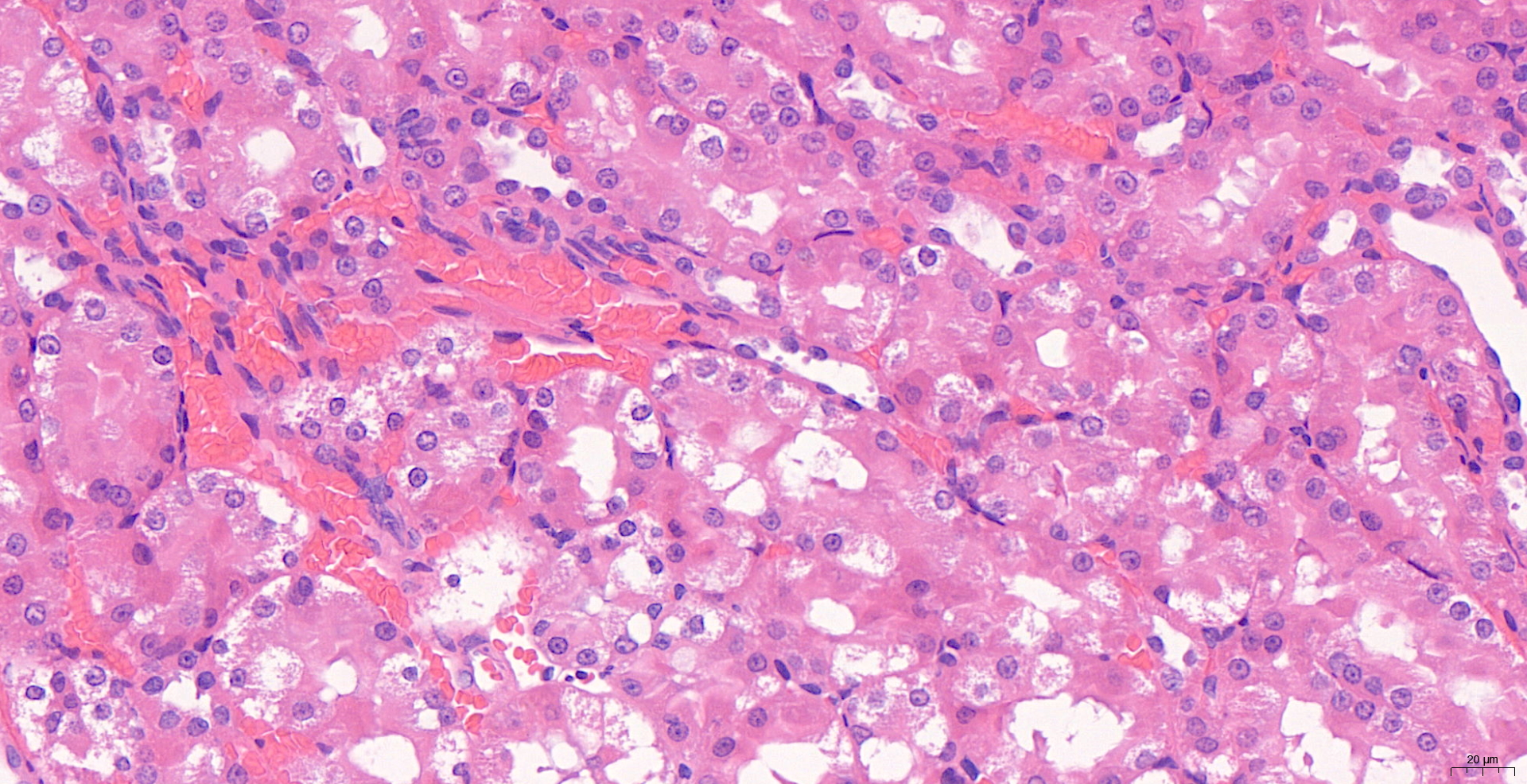


Prednisone


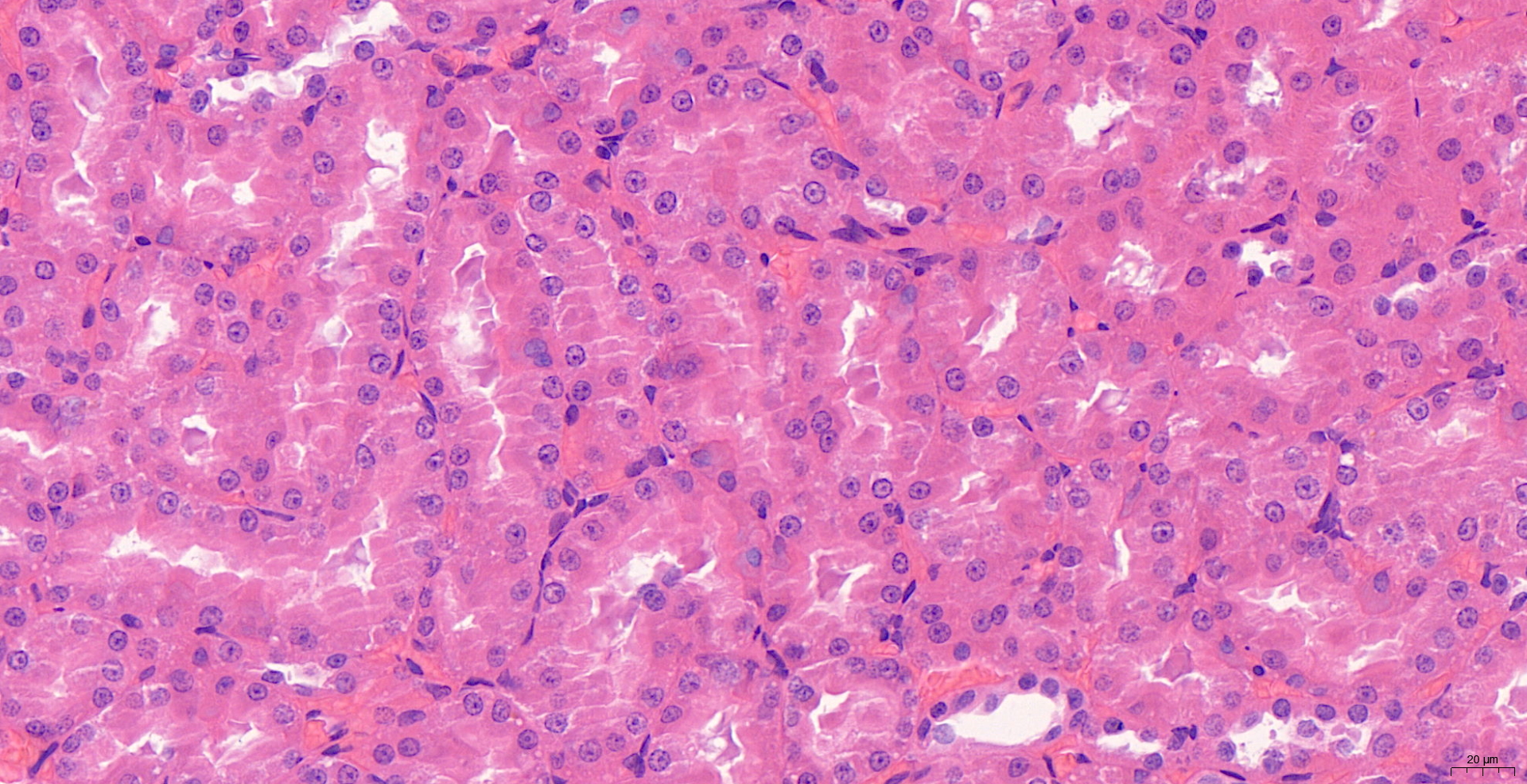


Icariin


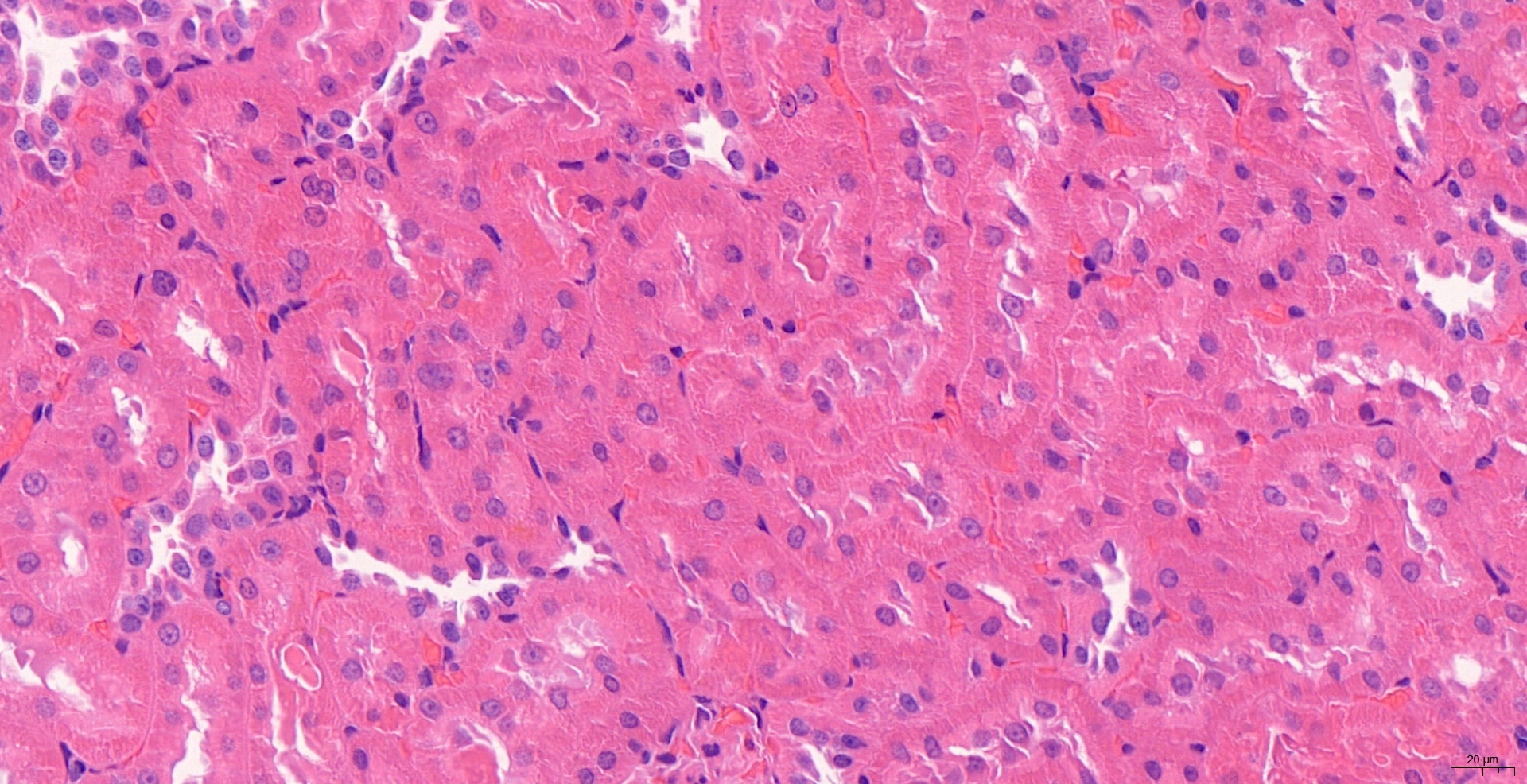


**Masson**

Control


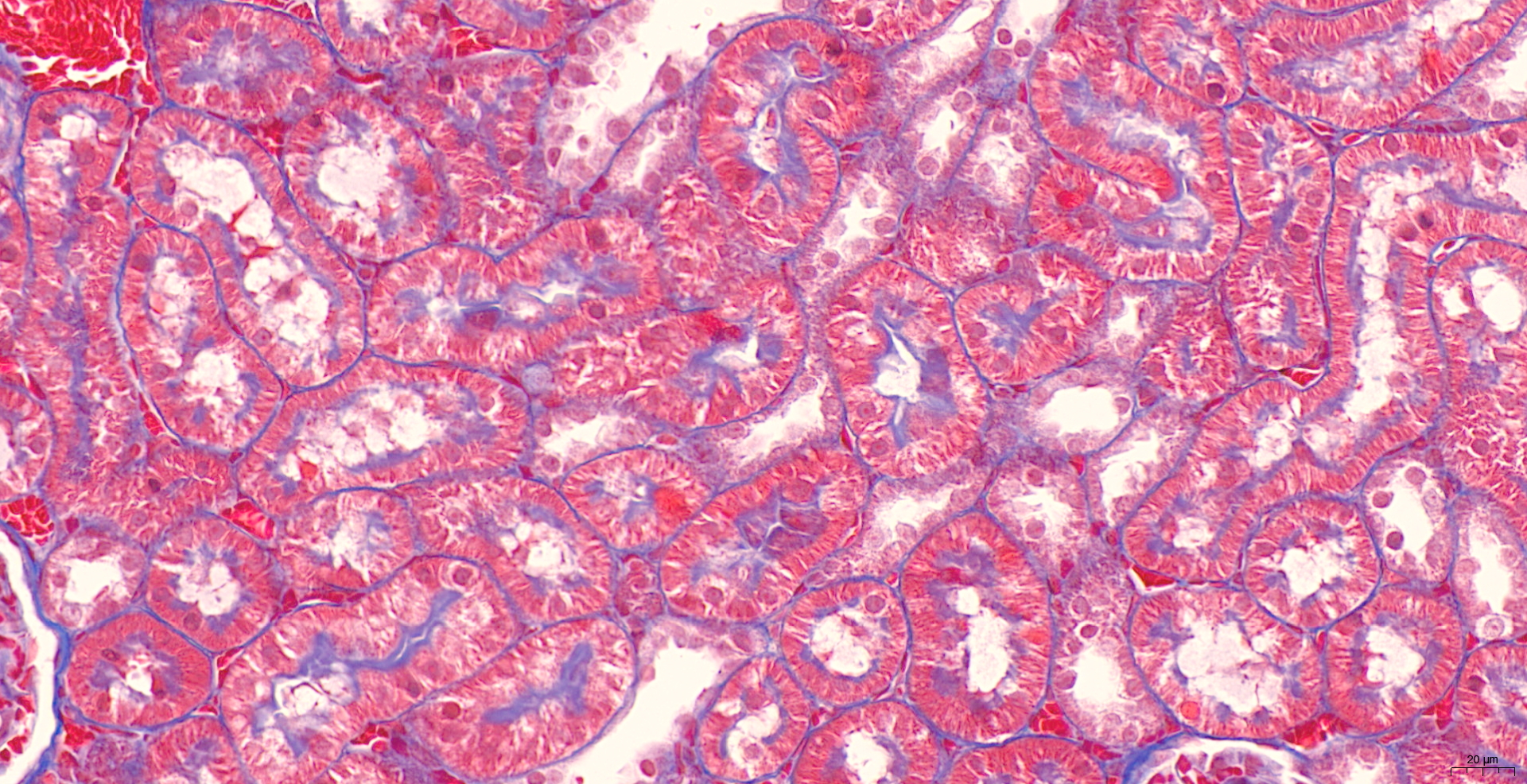


Doxorubicin


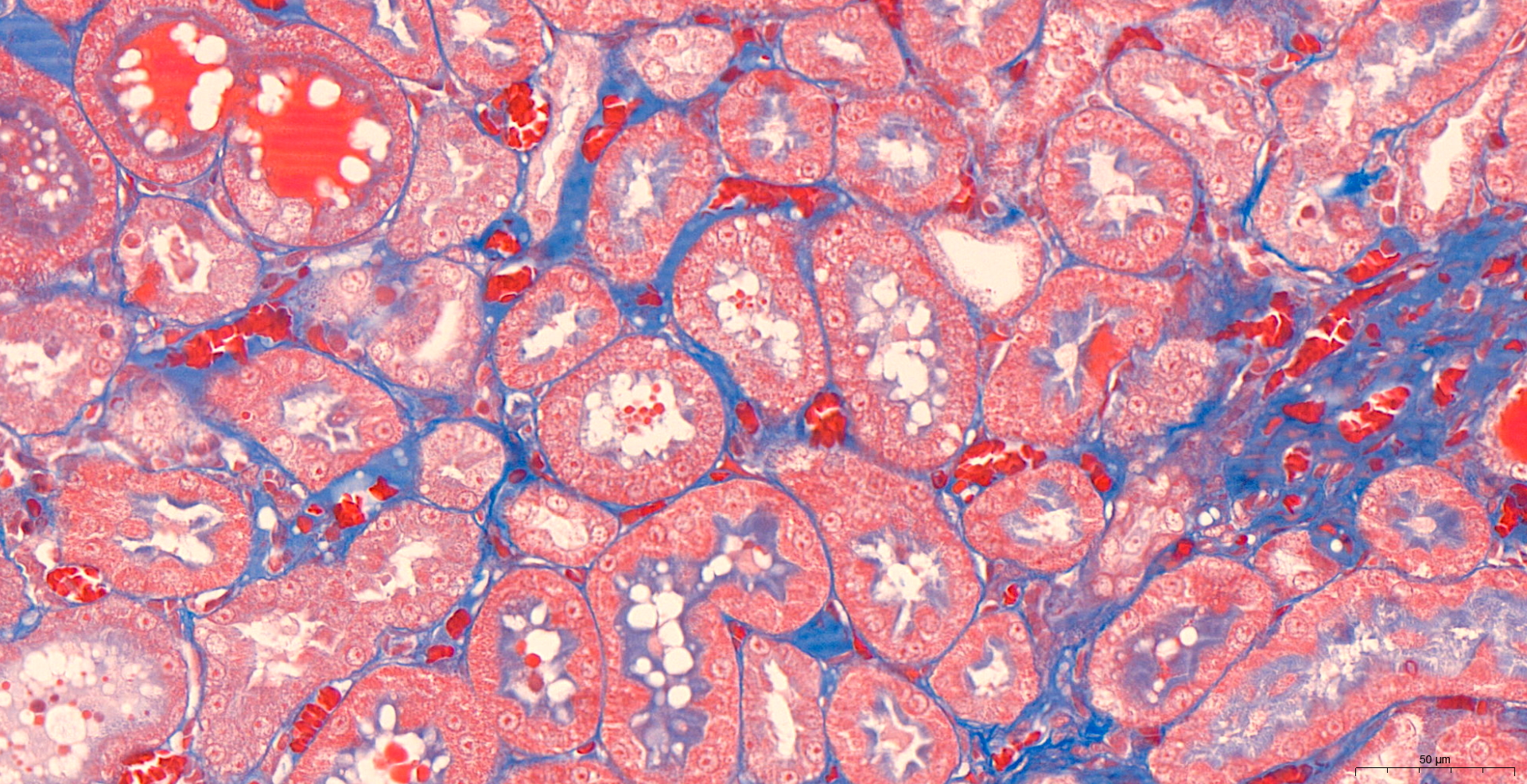


Prednisone


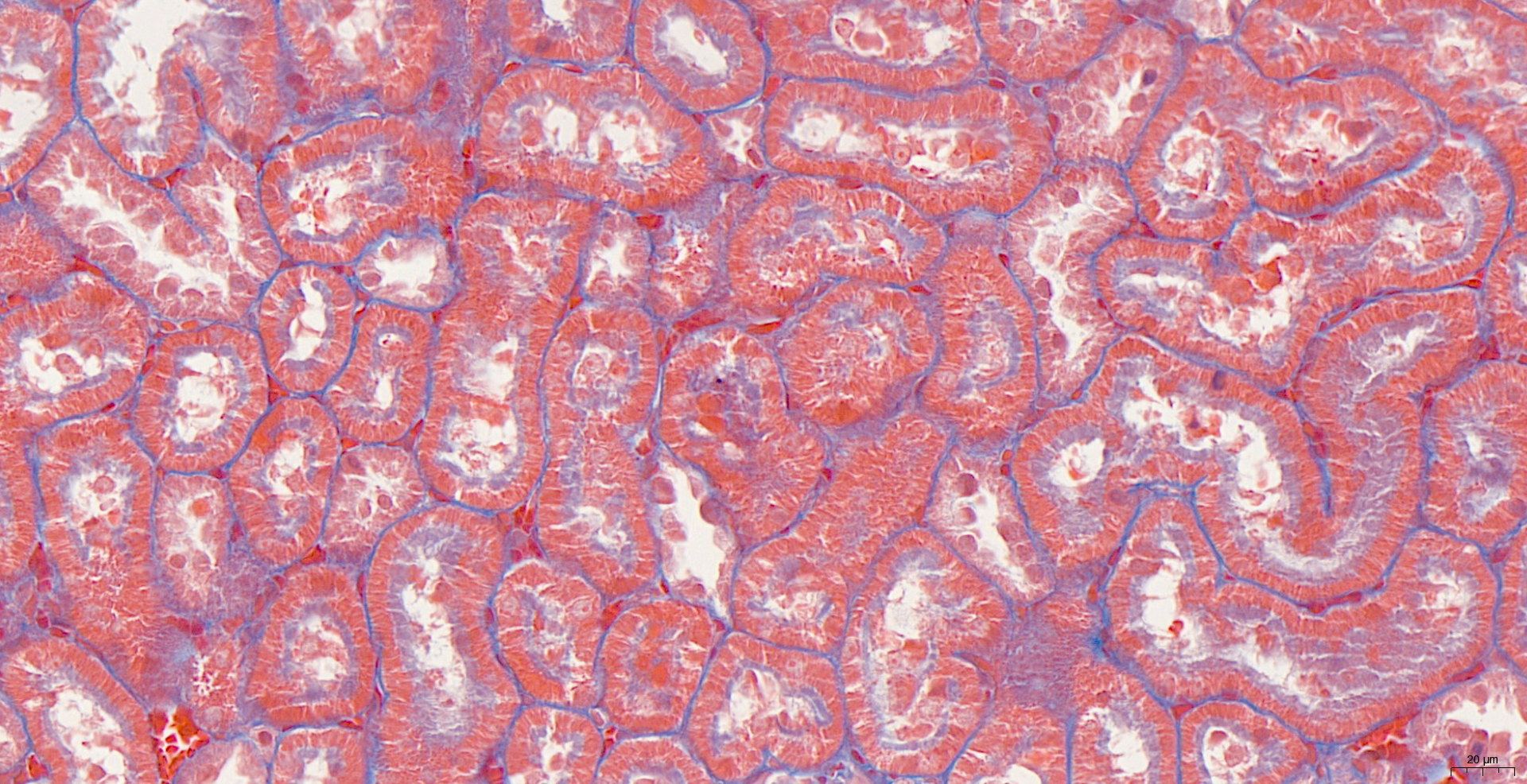


Icariin


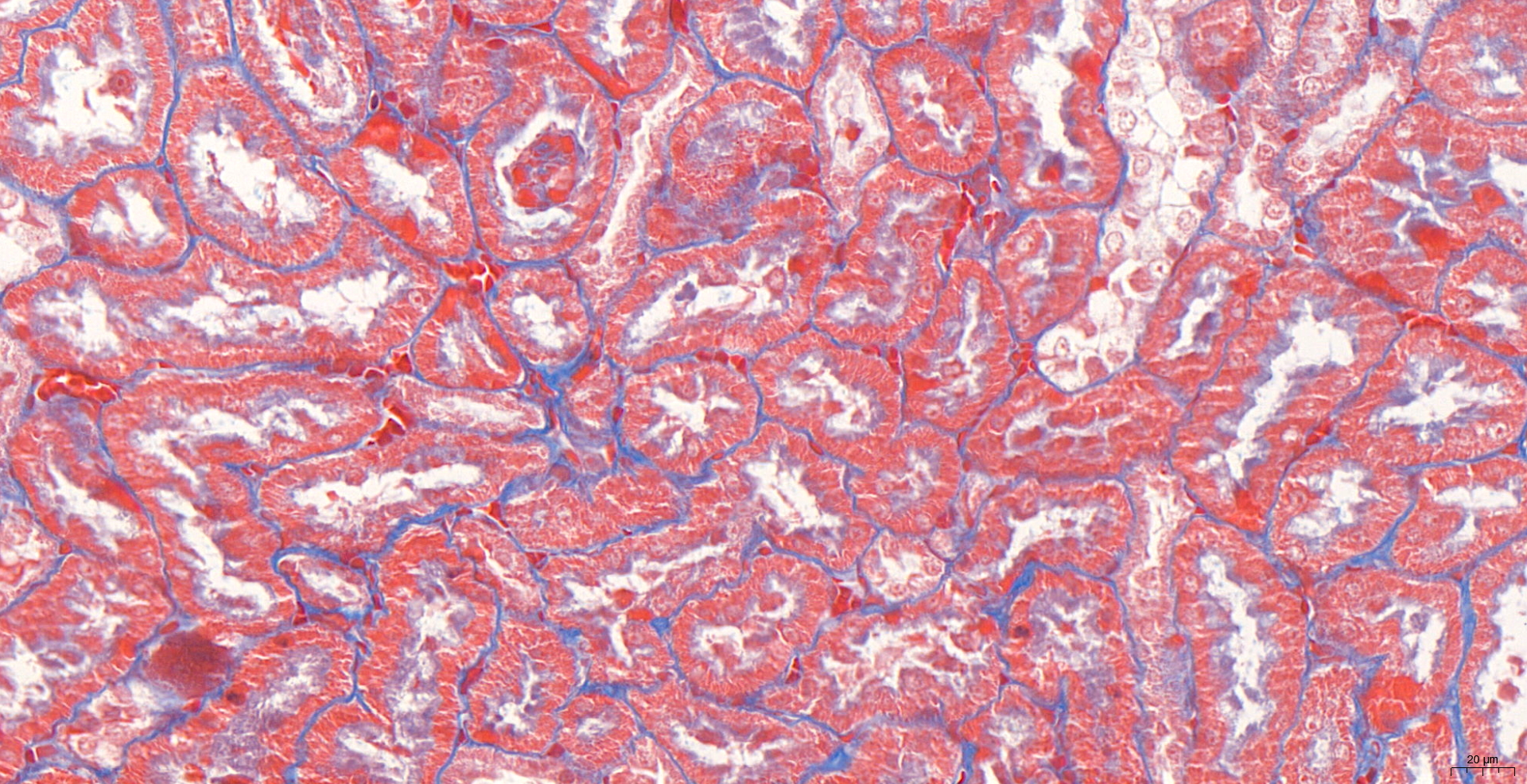


**TUNEL**

Control
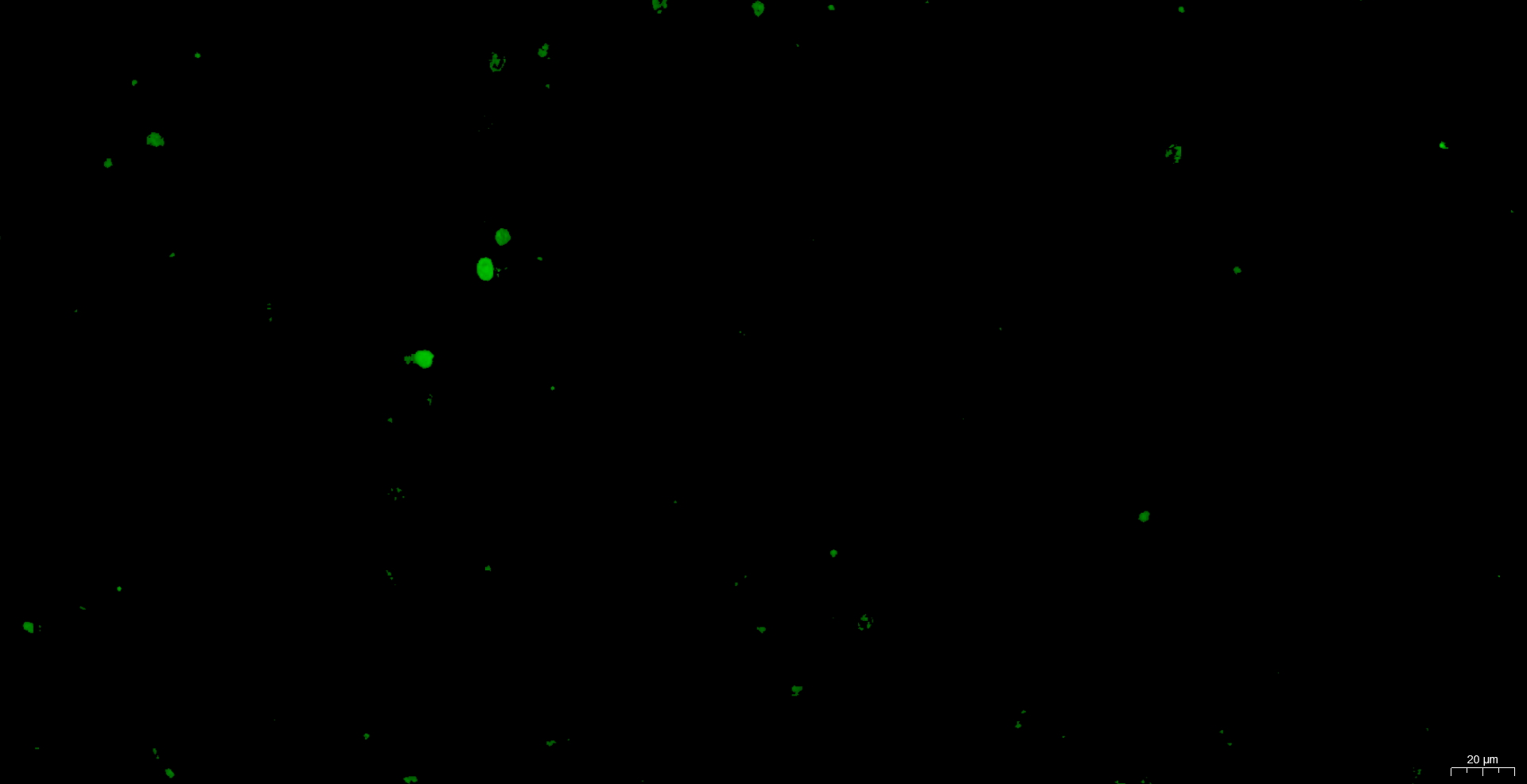


Doxorubicin
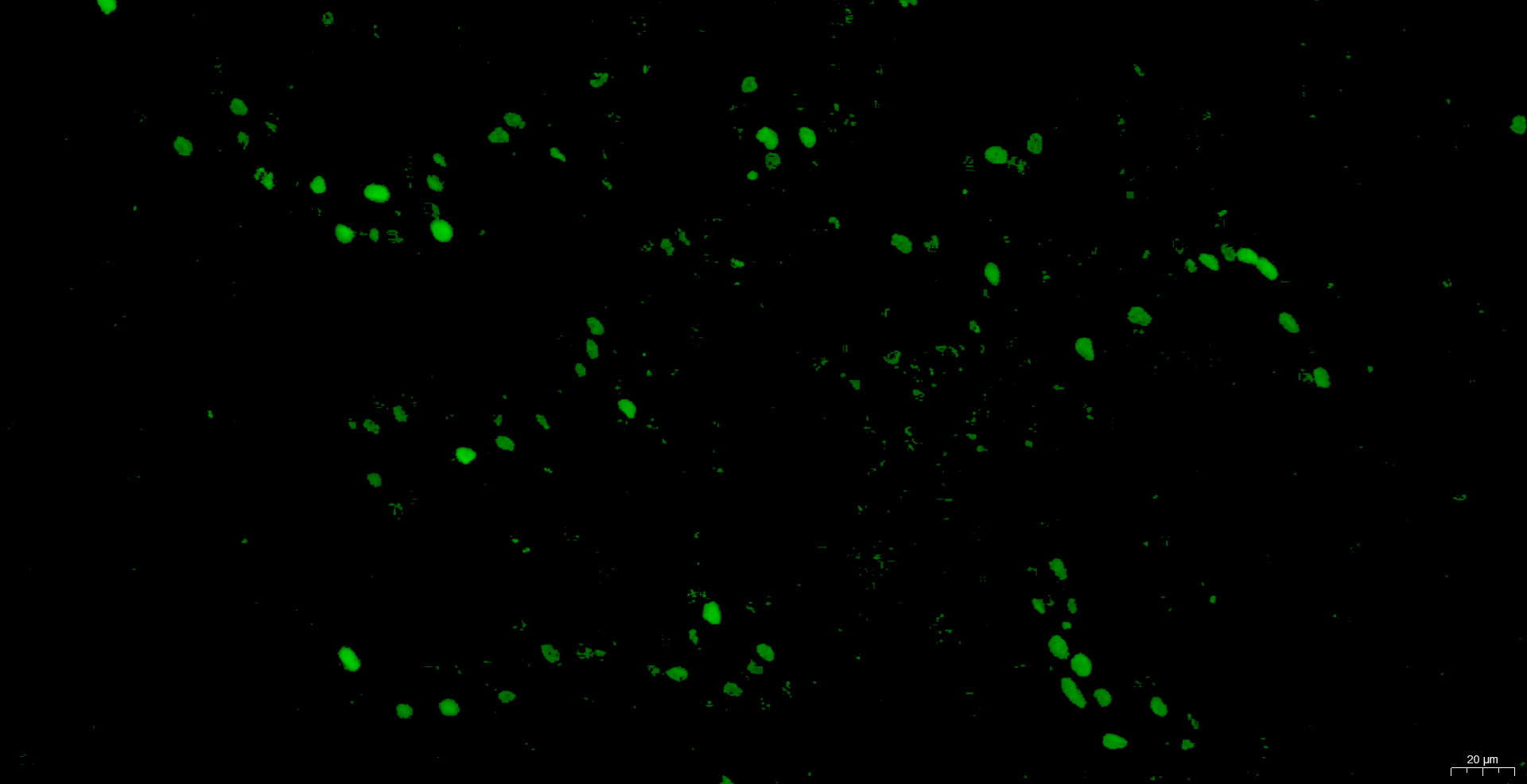


Prednisone
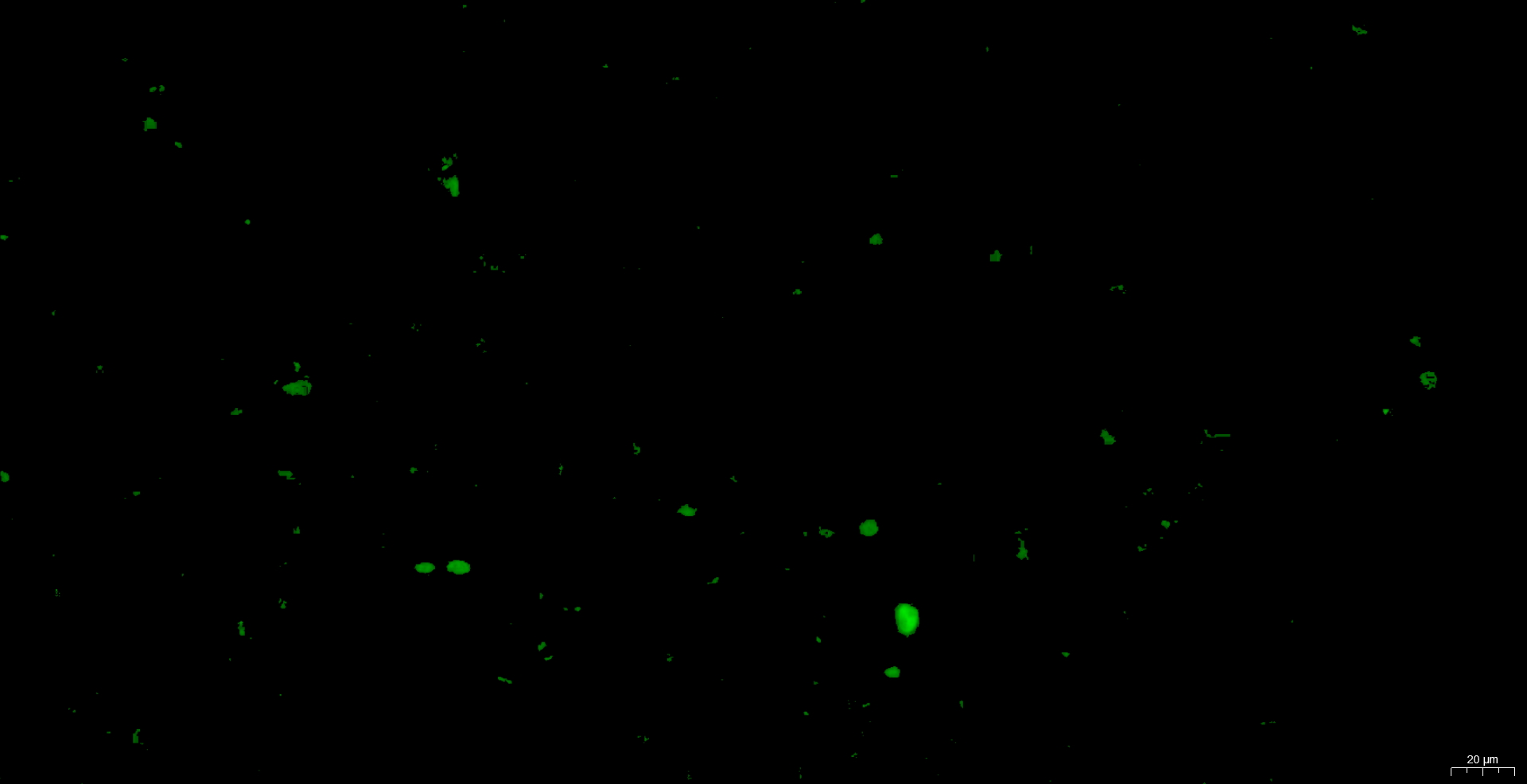


Icariin


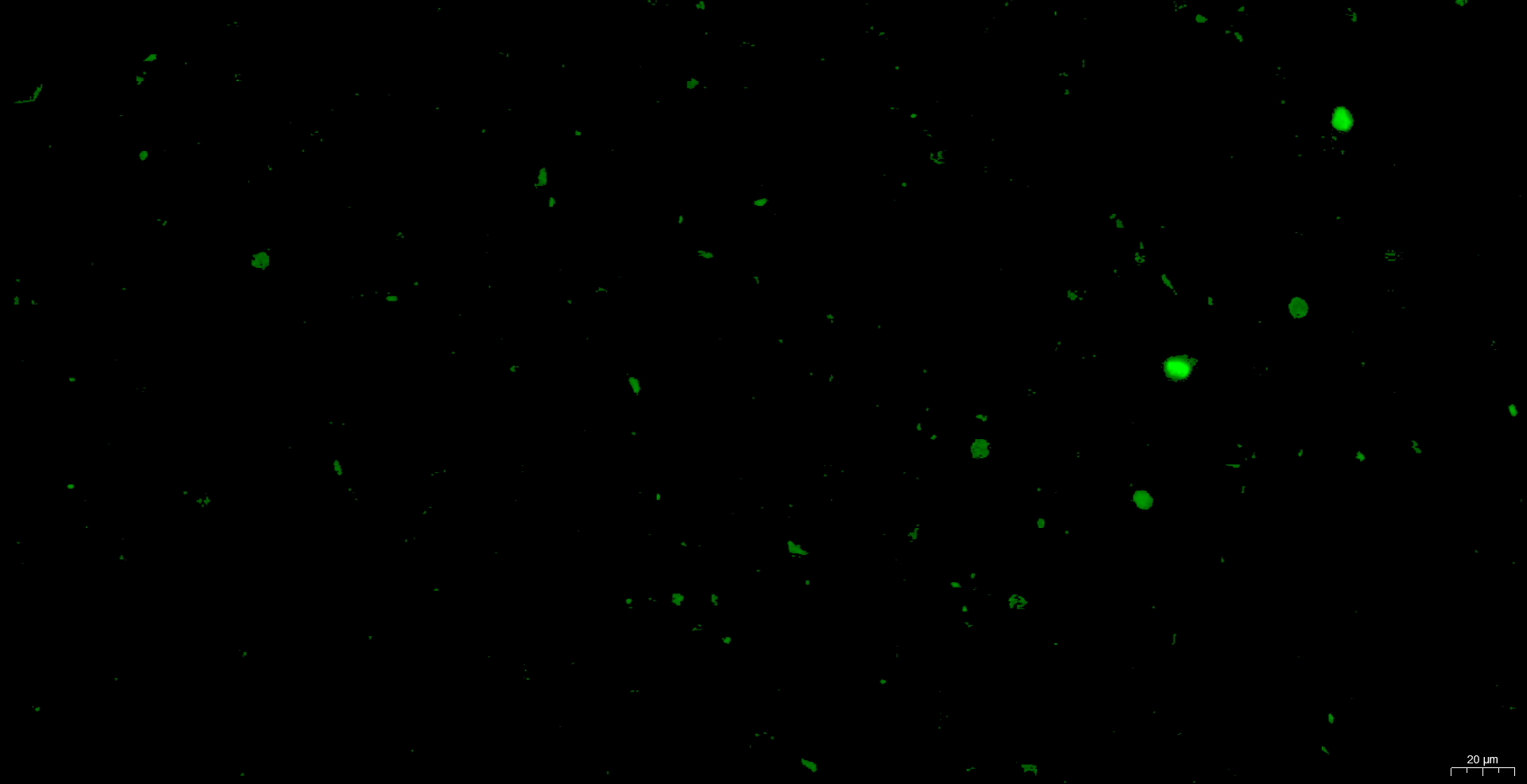


**DAPI**

Control
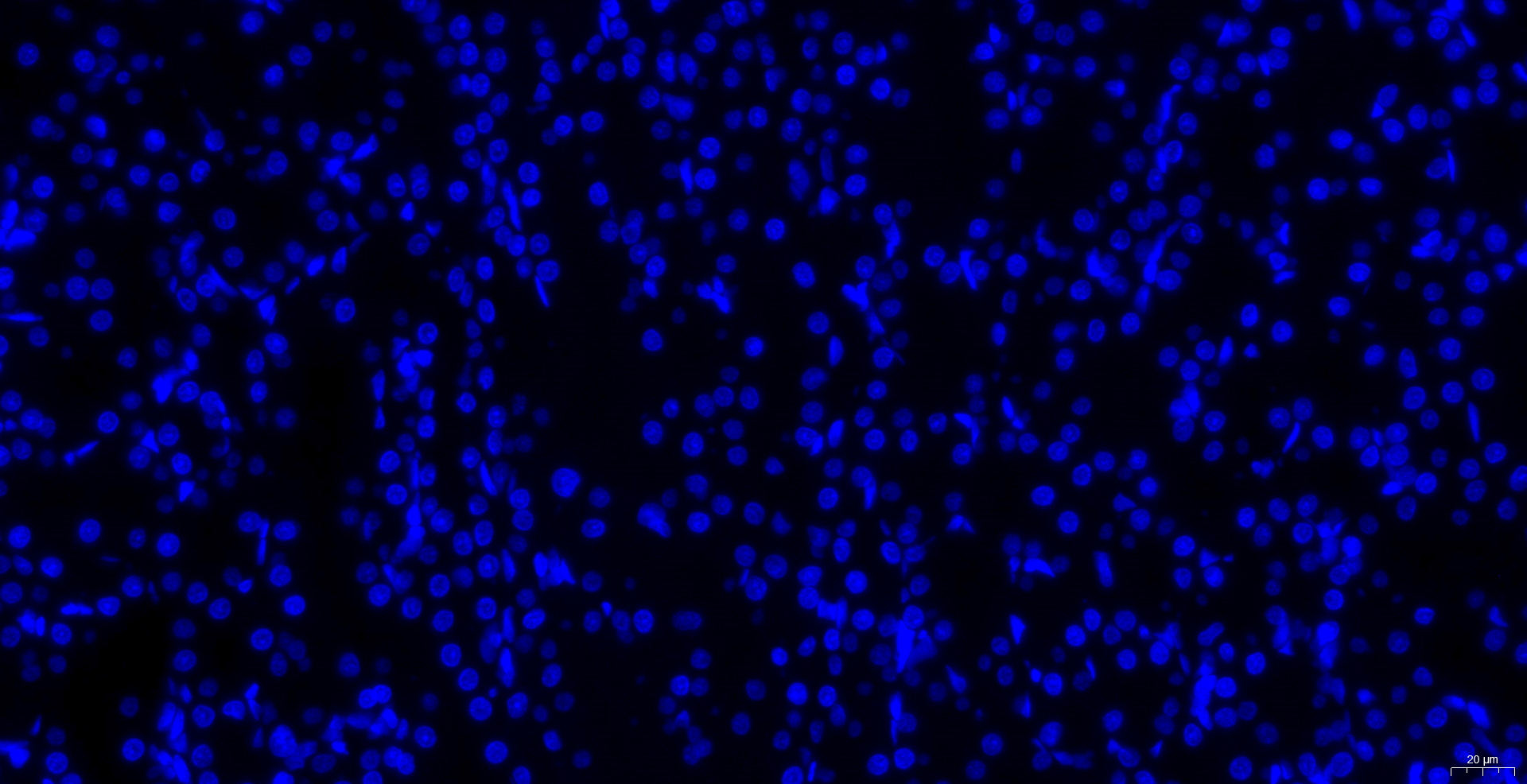


Doxorubicin
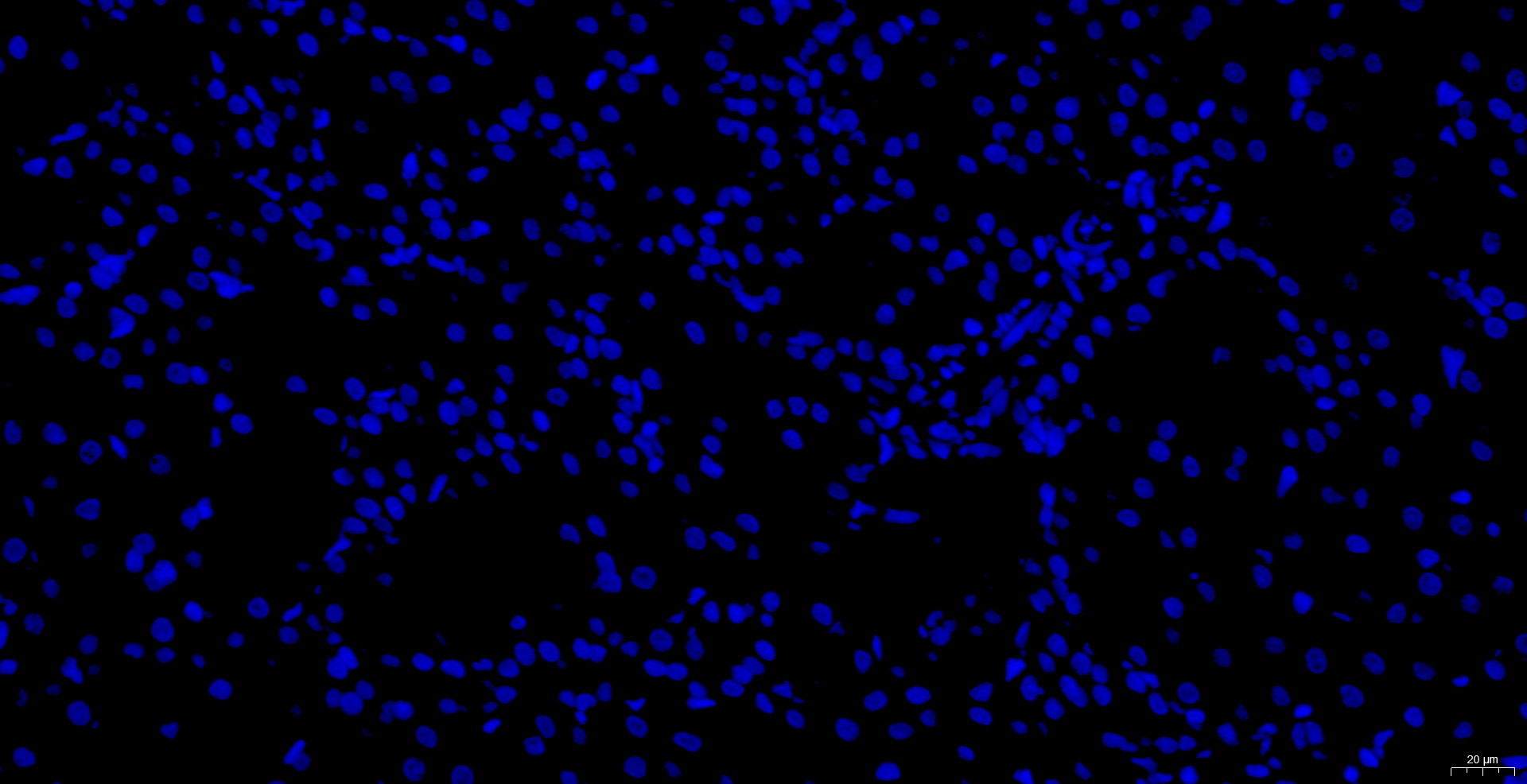


Prednisone
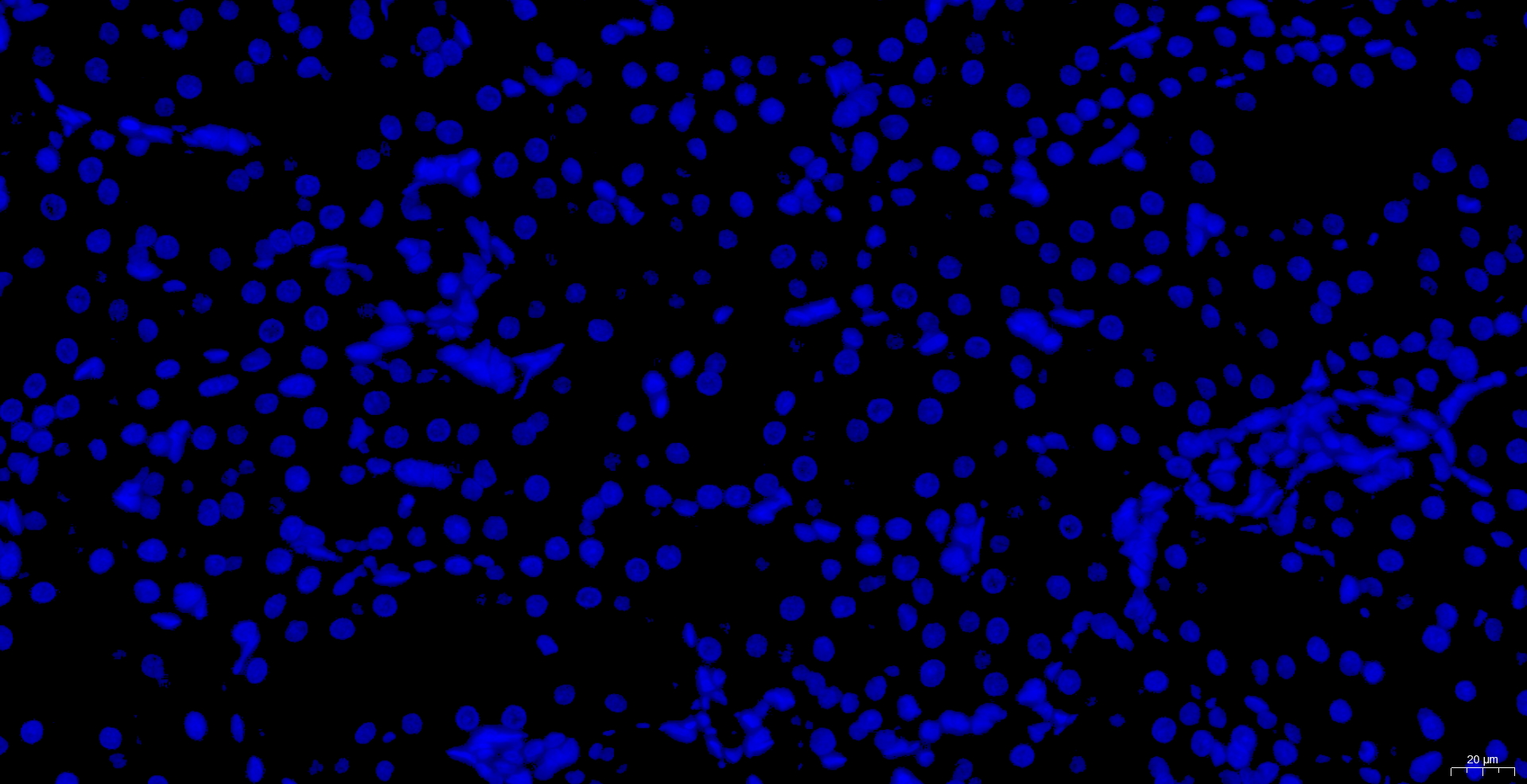


Icariin
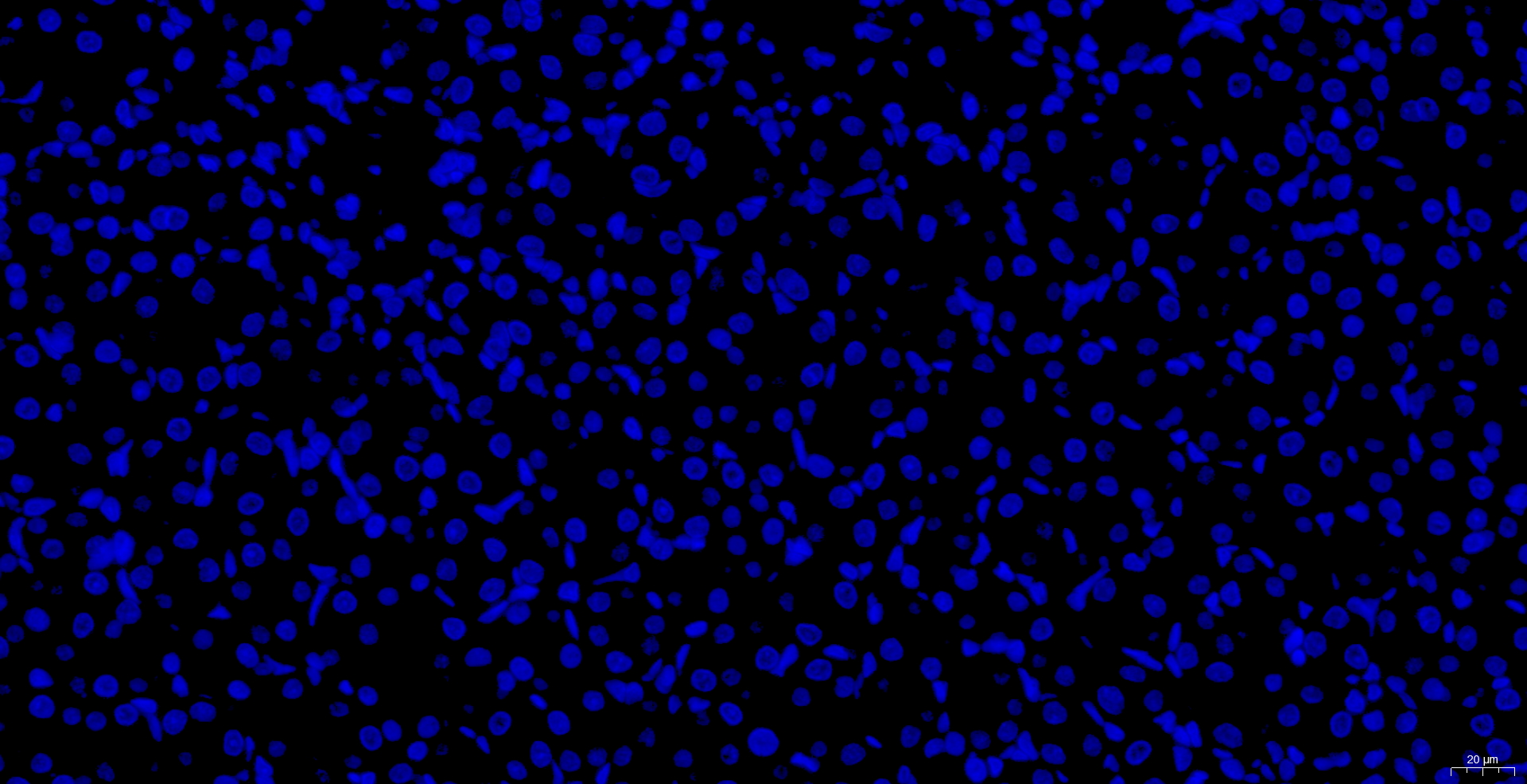


**Merge**

Control
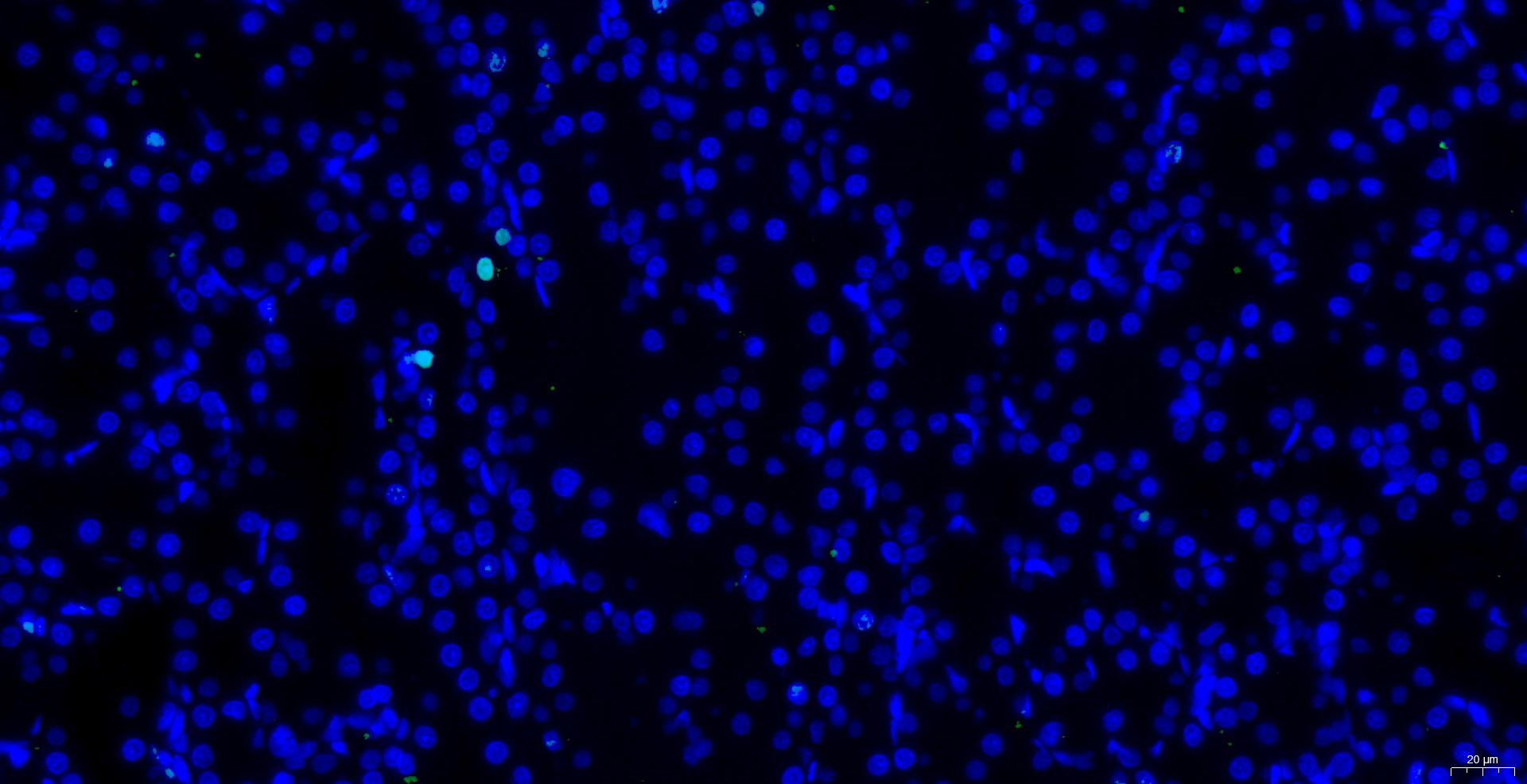


Doxorubicin
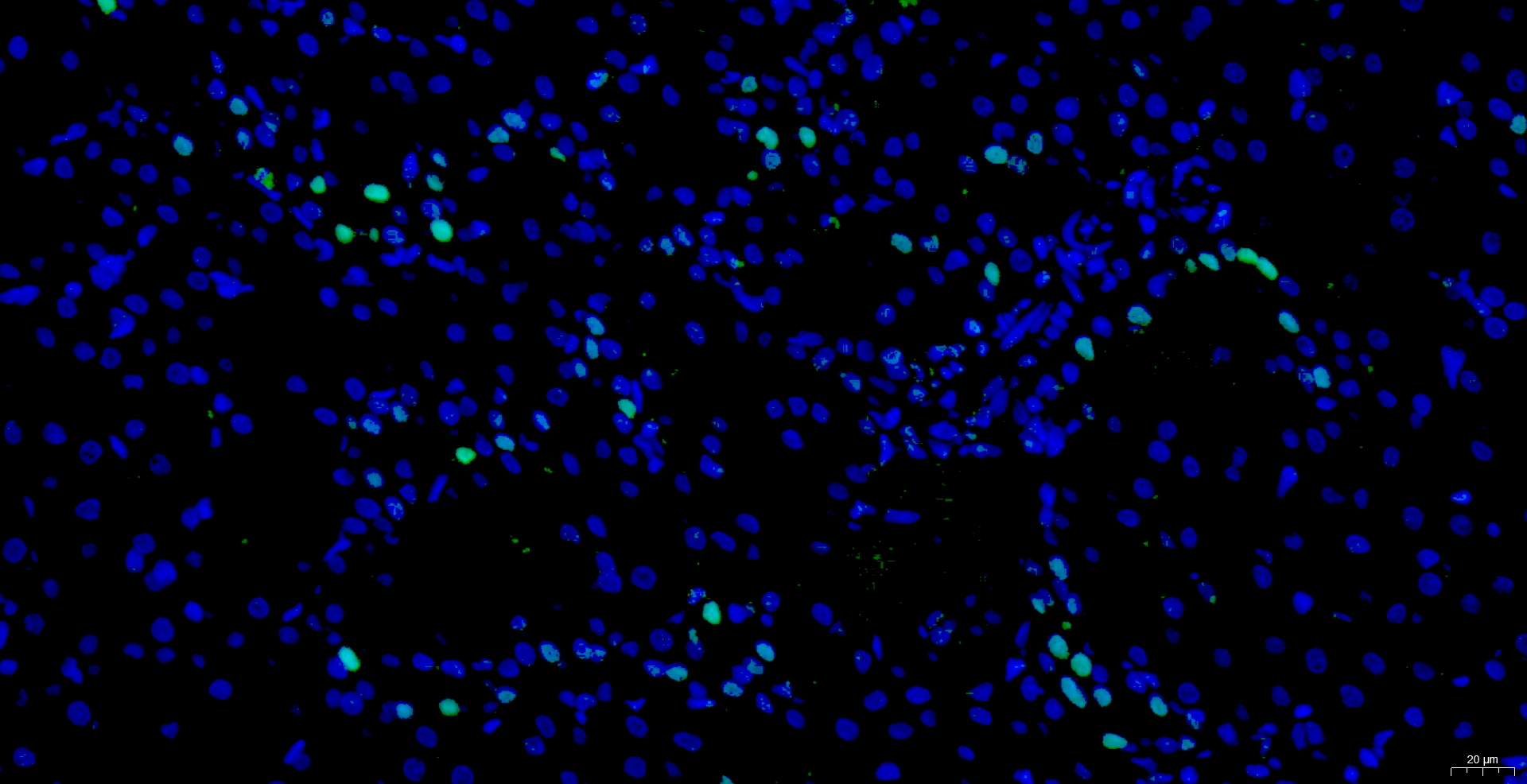


Prednisone
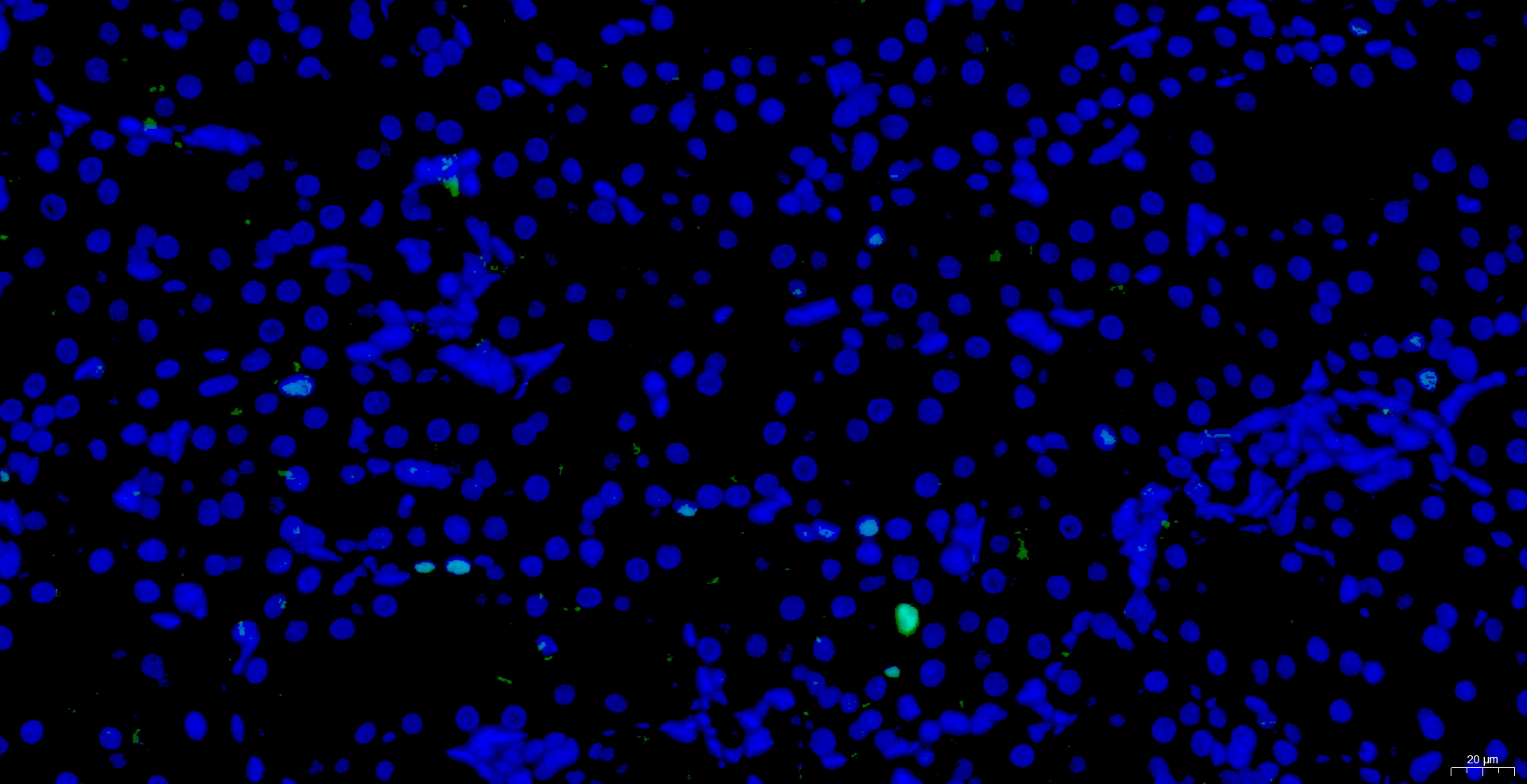


Icariin
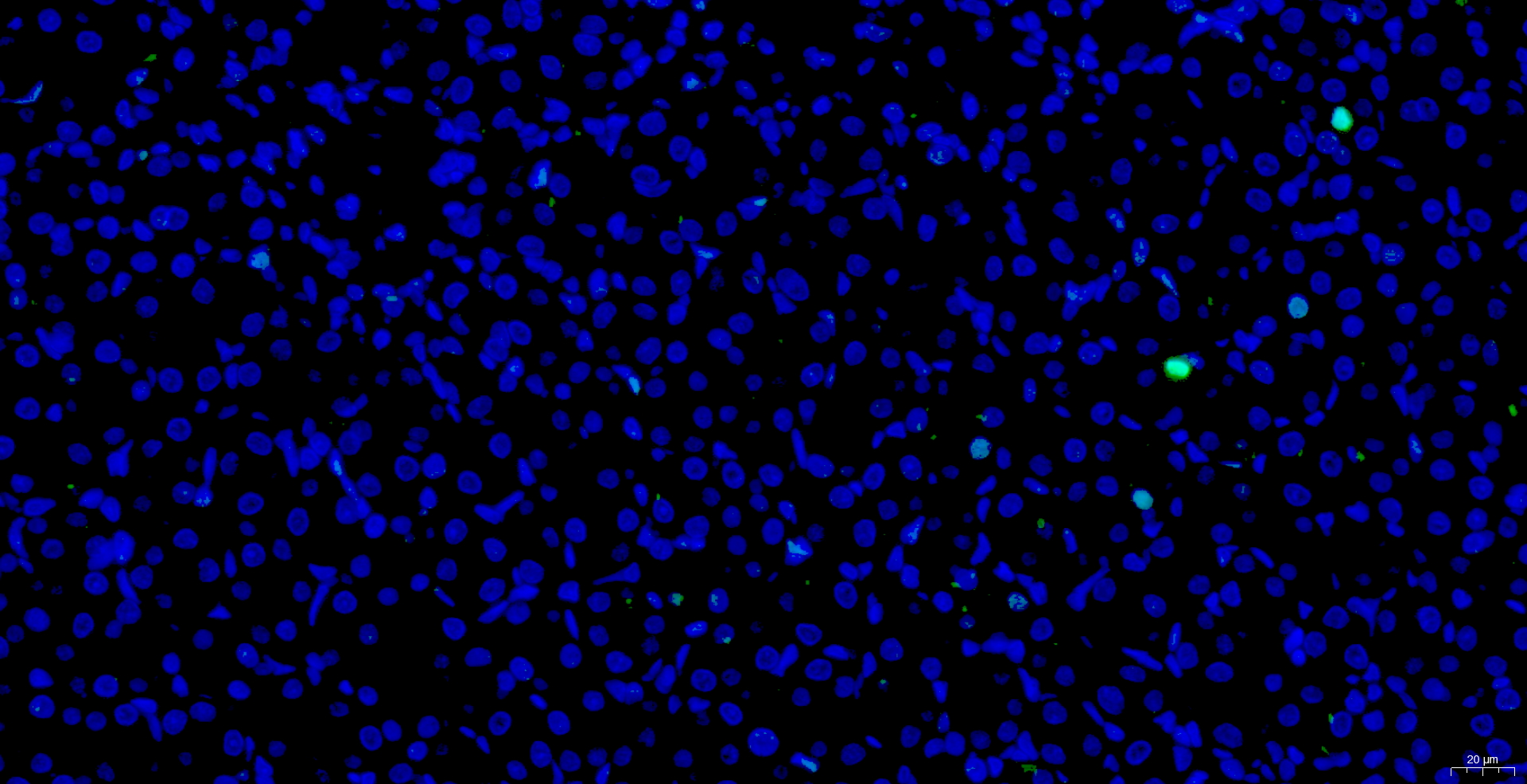


**Immunohistochemistry(E-cadherin)**

Control
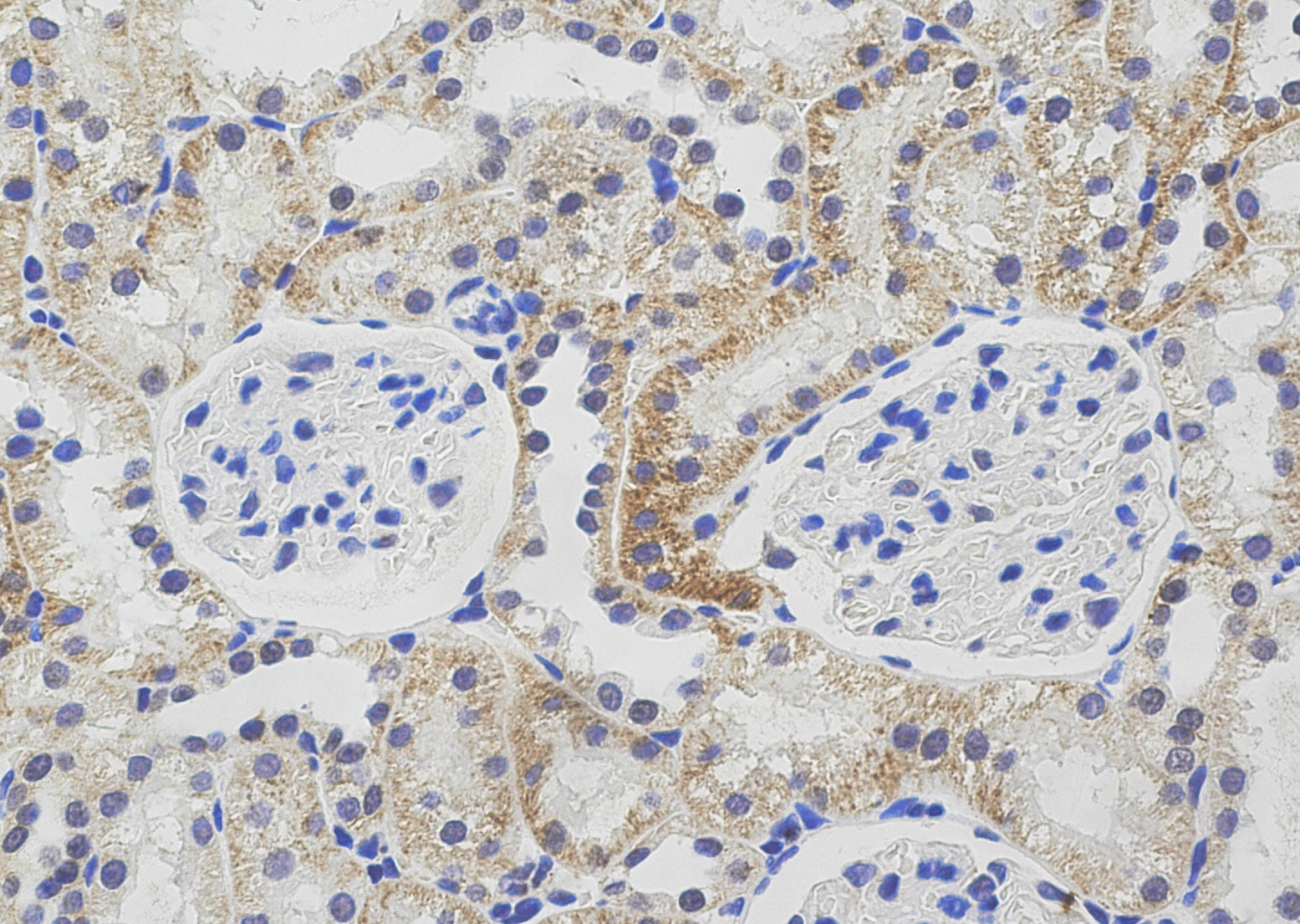


Doxorubicin
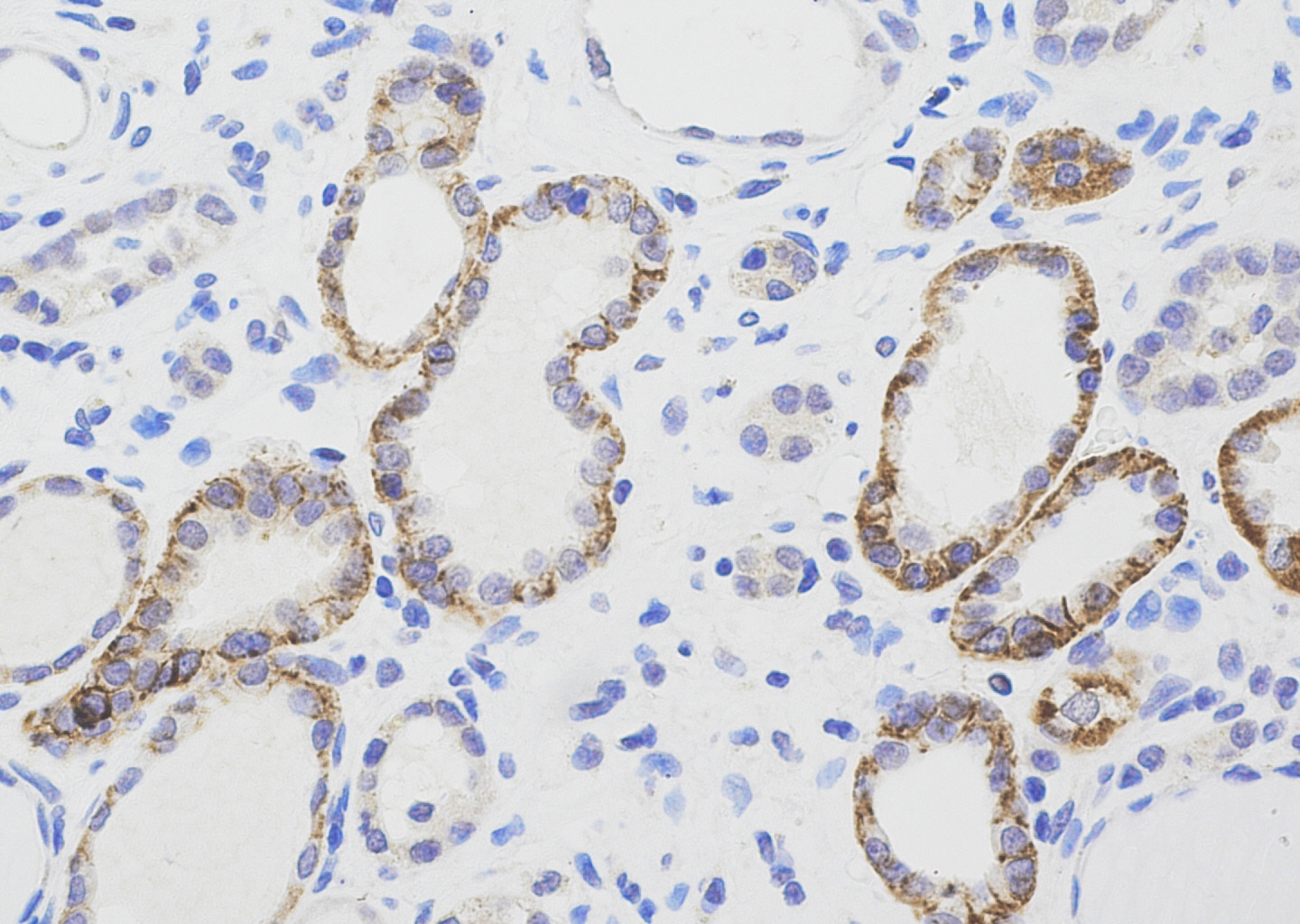


Prednisone
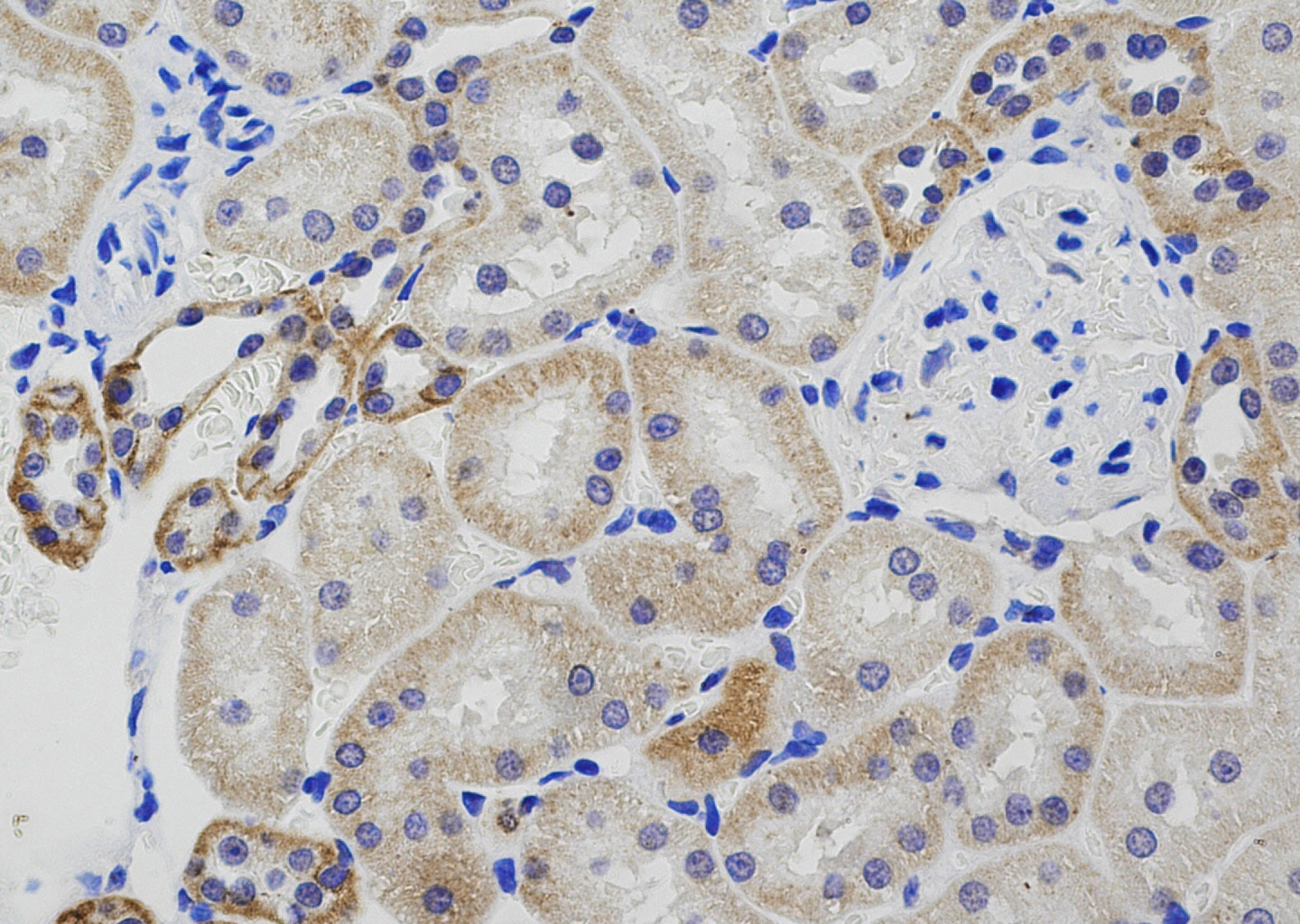


Icariin


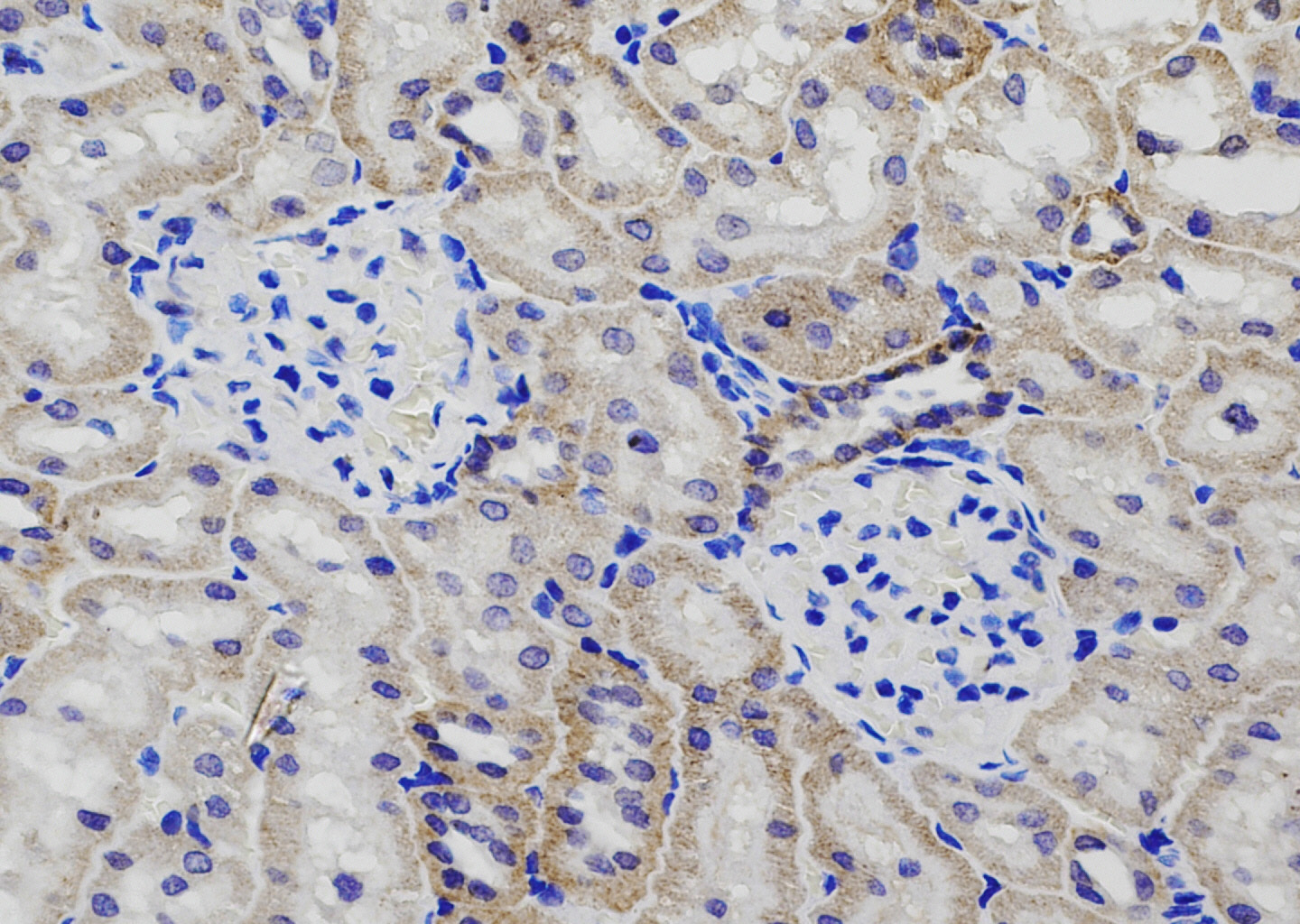


**Immunohistochemistry(α-SMA)**

Control
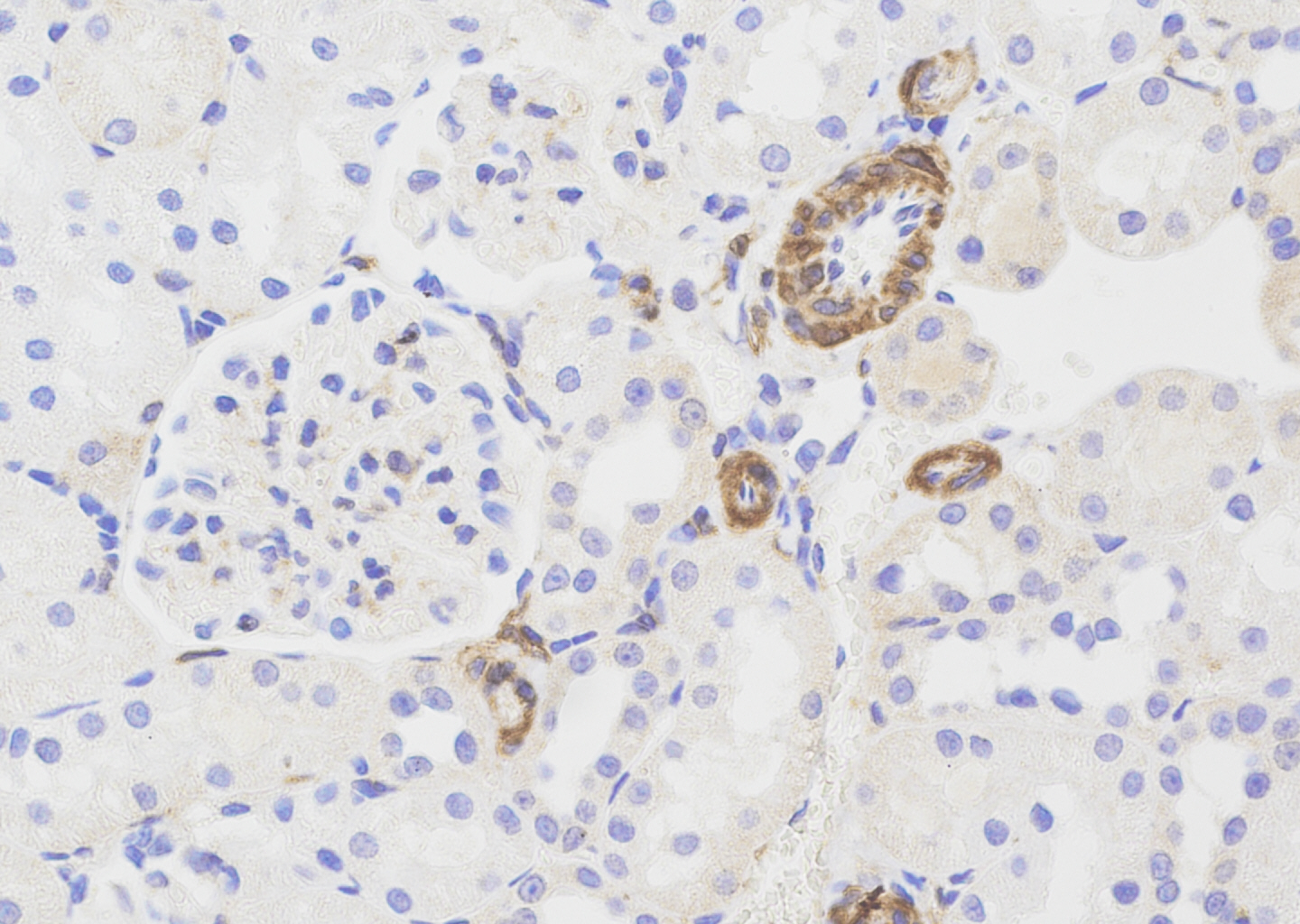


Doxorubicin
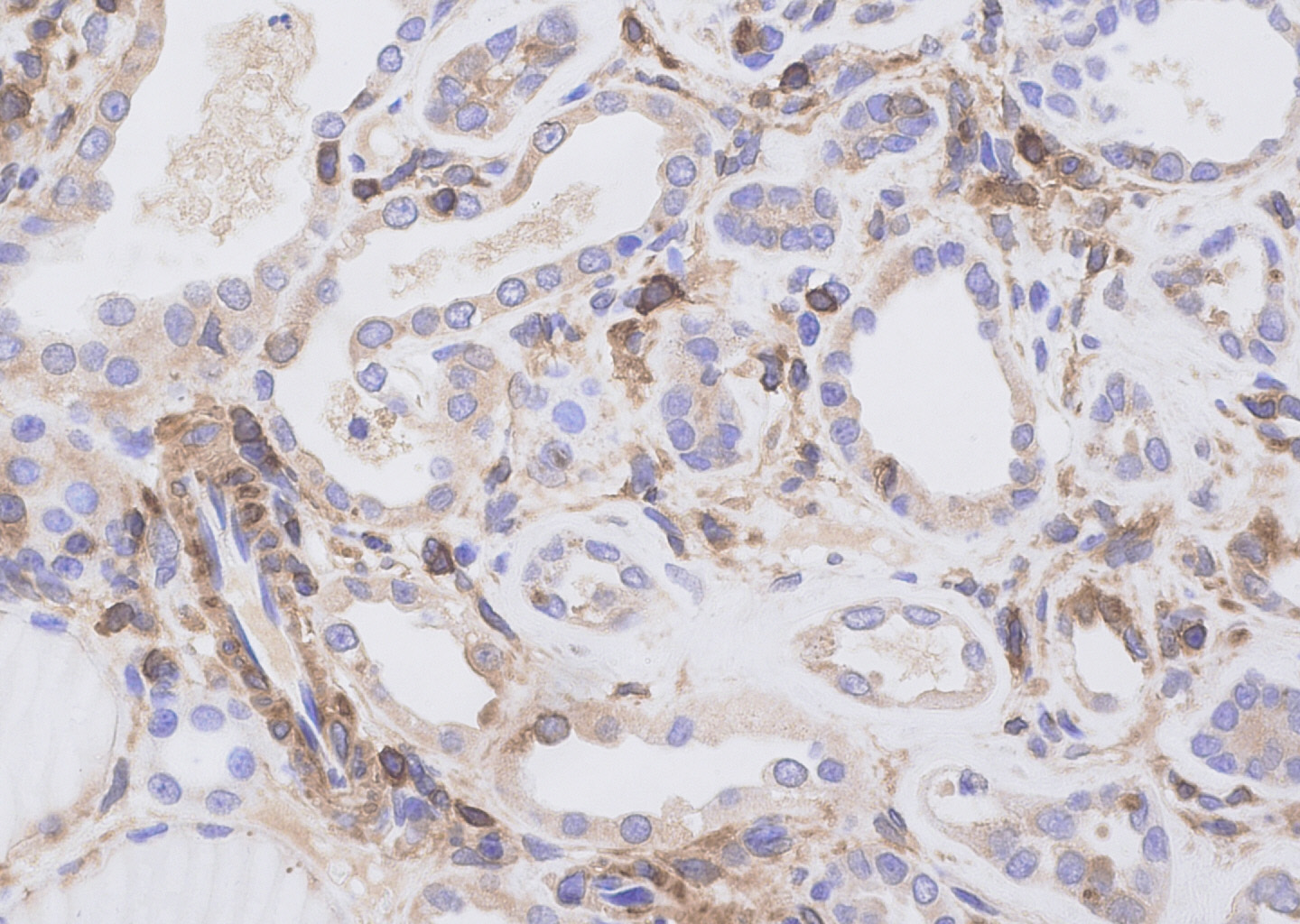


Prednisone
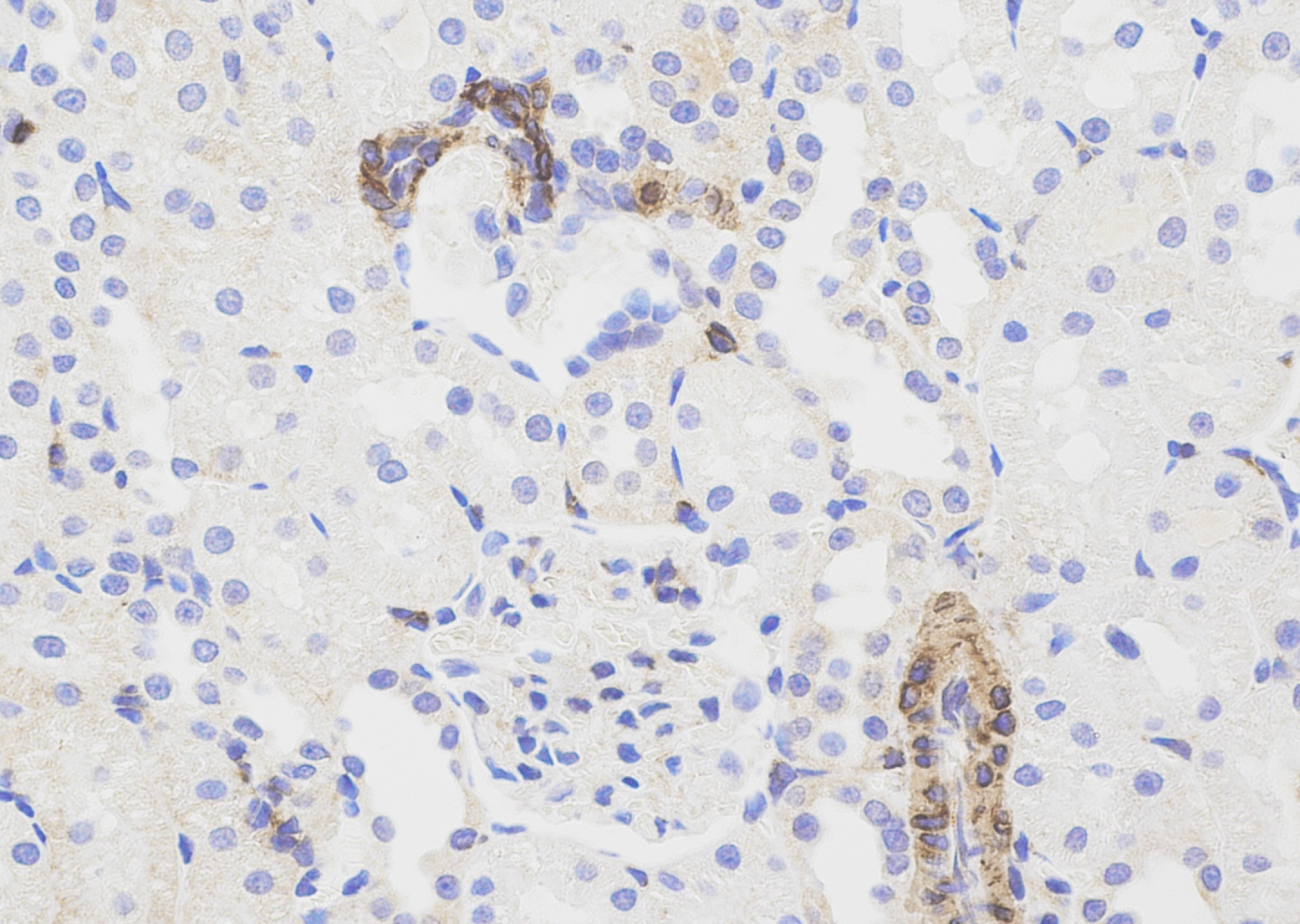


Icariin


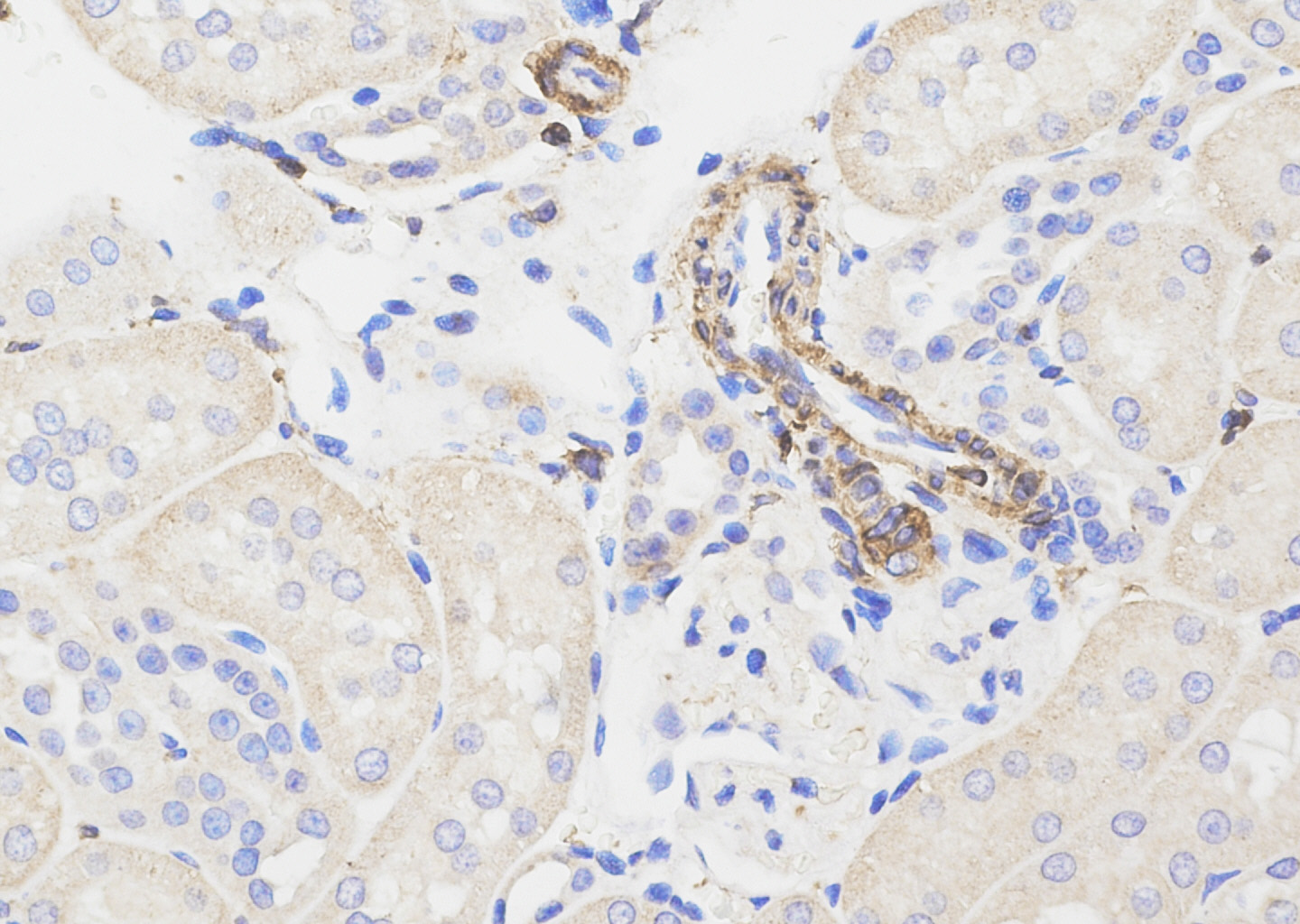


**WB**

**Caspase-1**


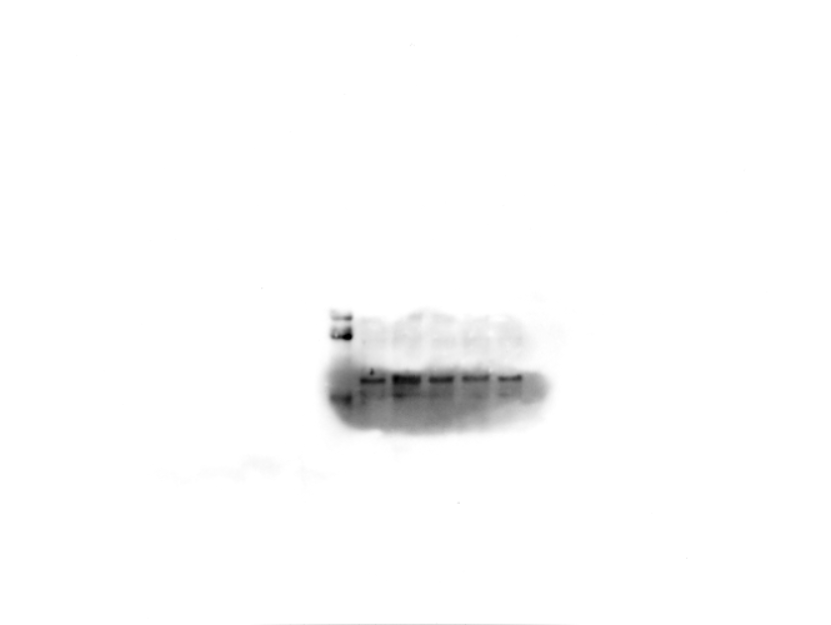


**IL-1β**

**
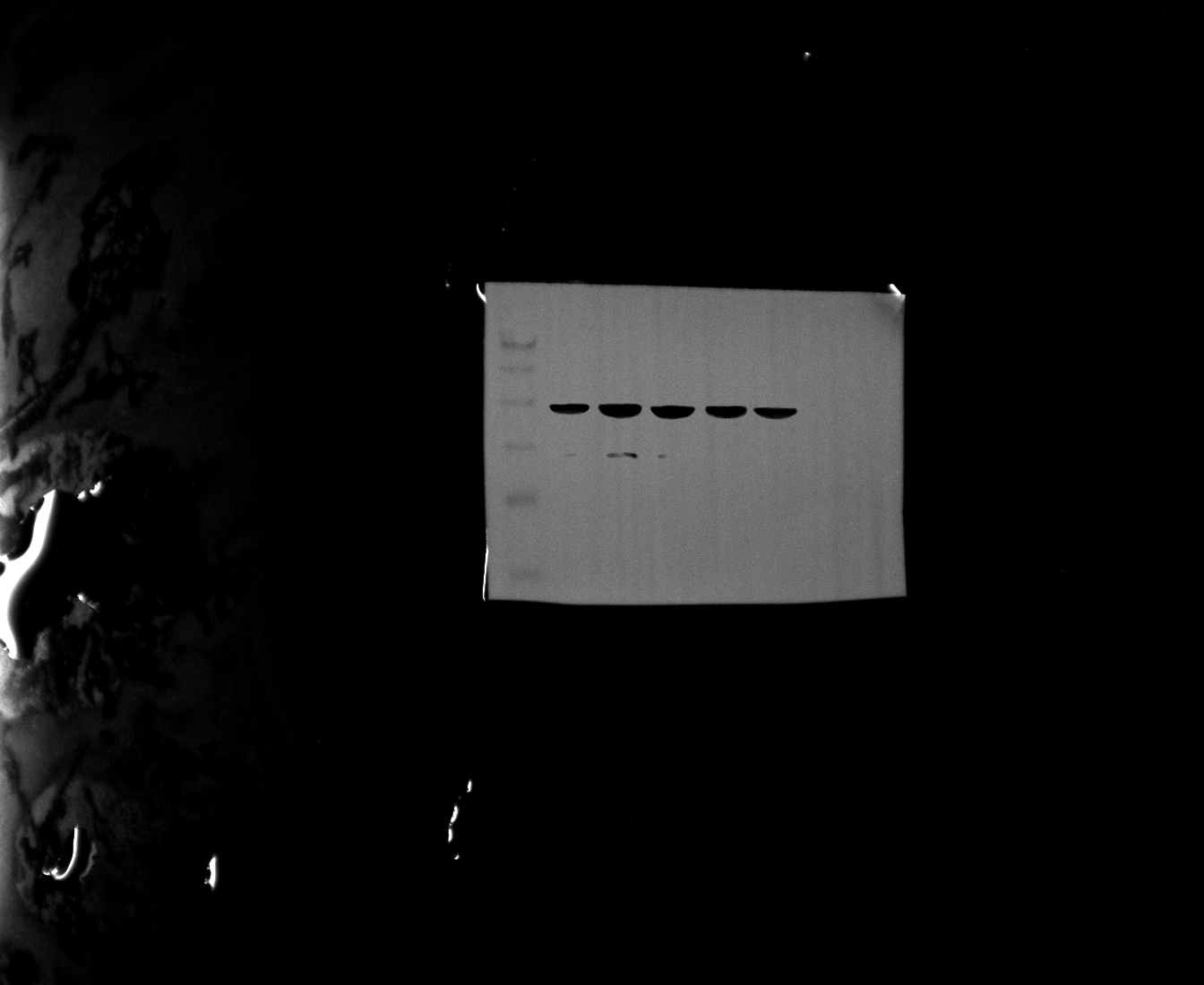
**

**NLRP3**


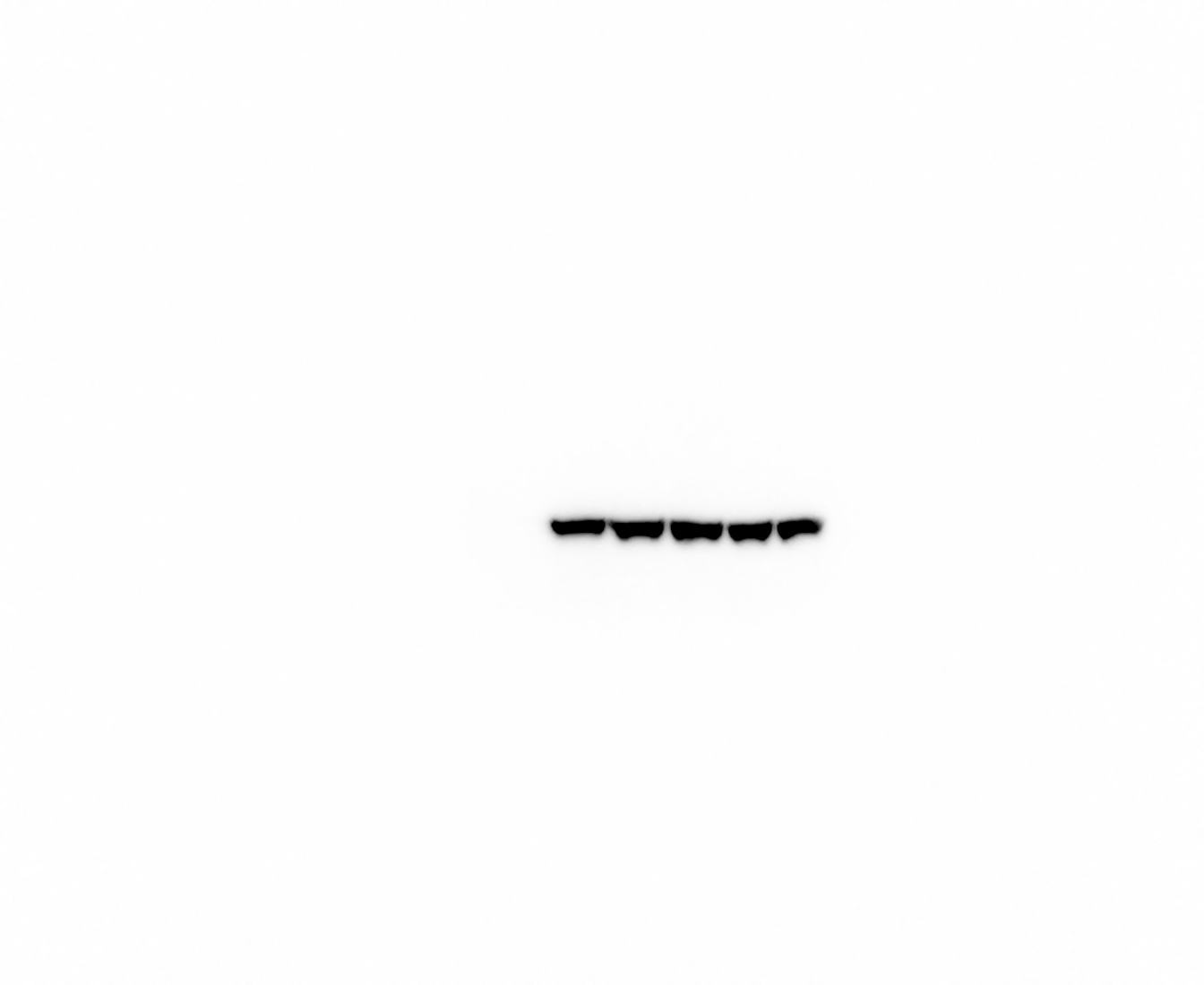


**GSDMD**


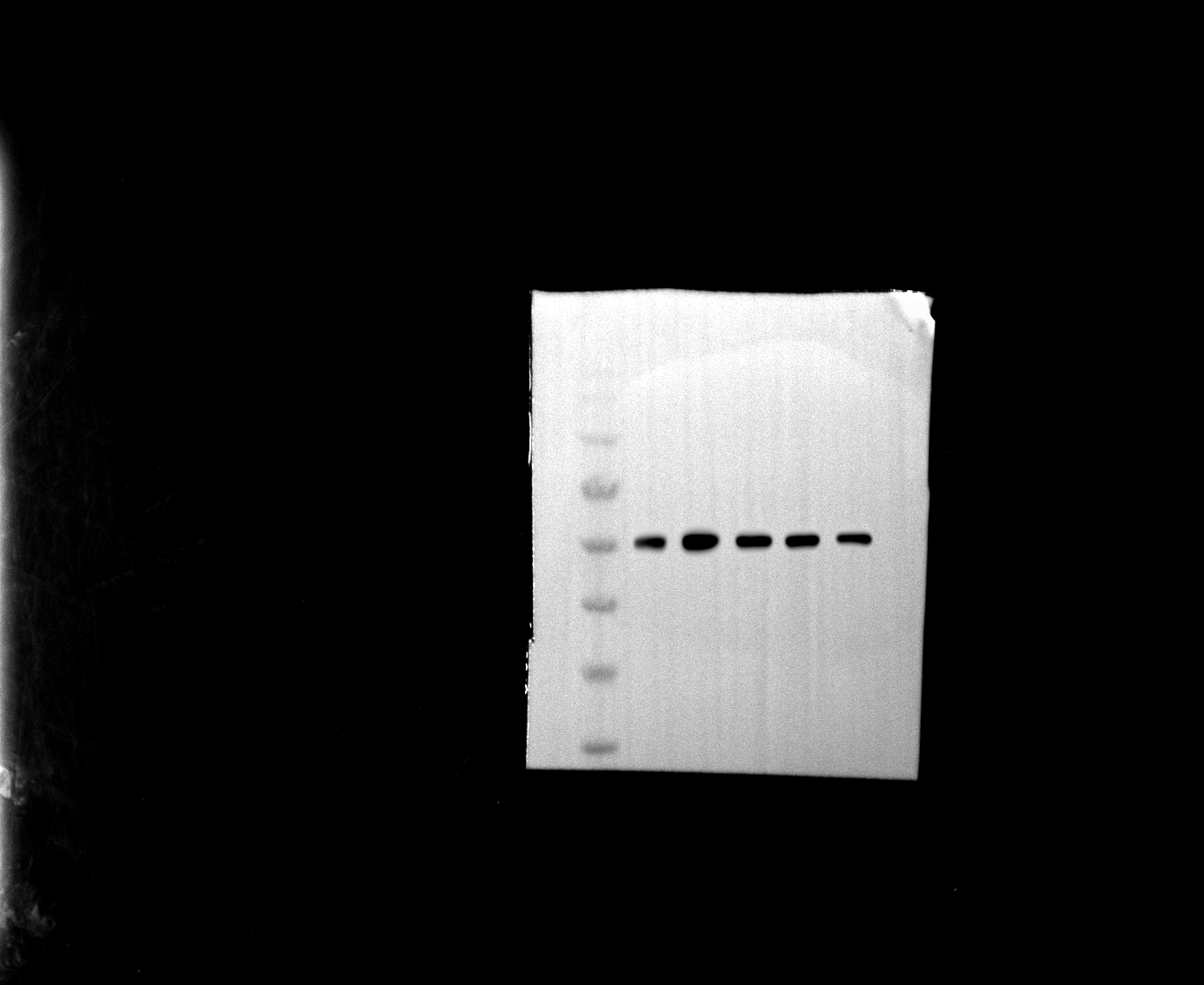


**E-cadherin**


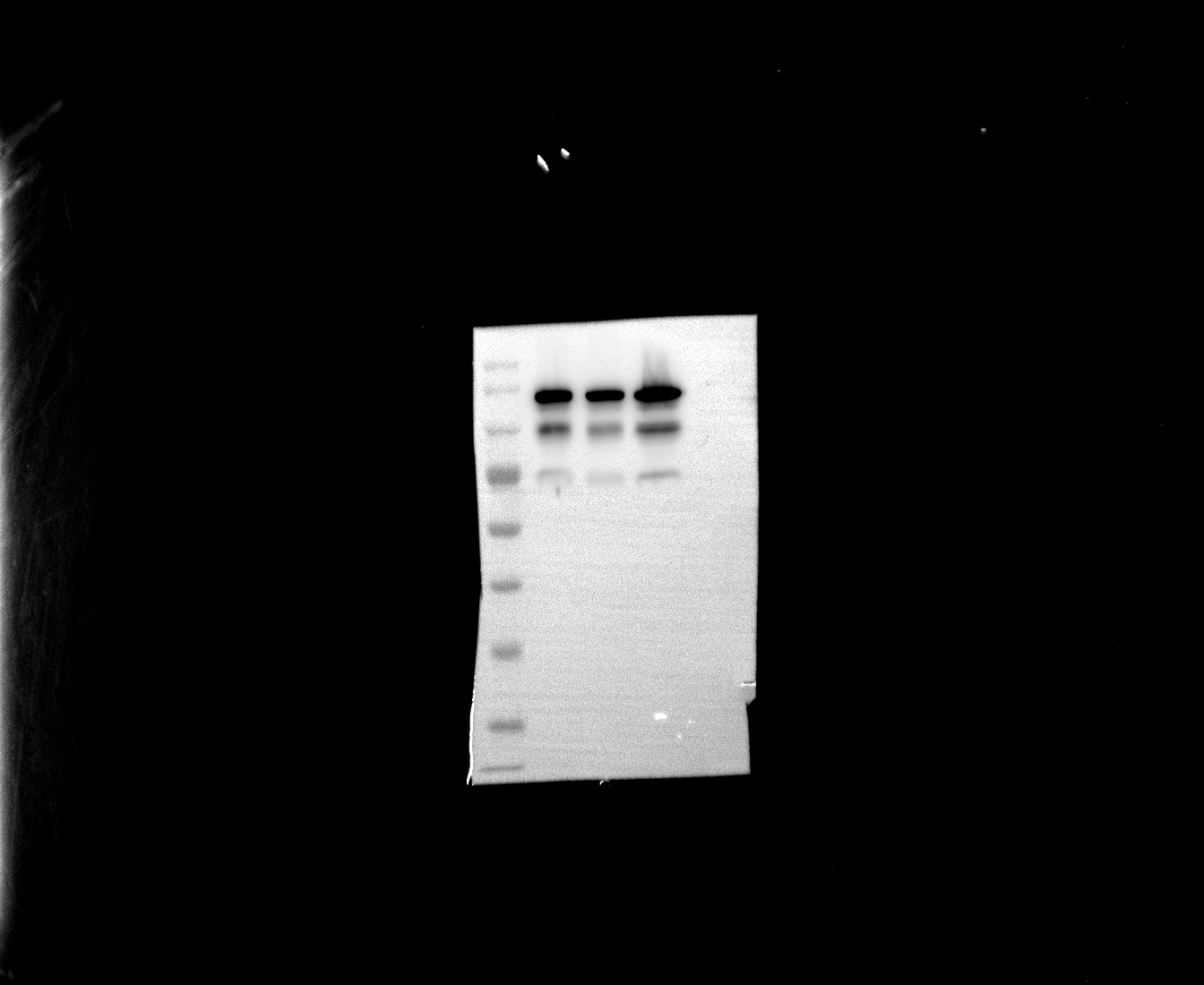


**α-SMA**


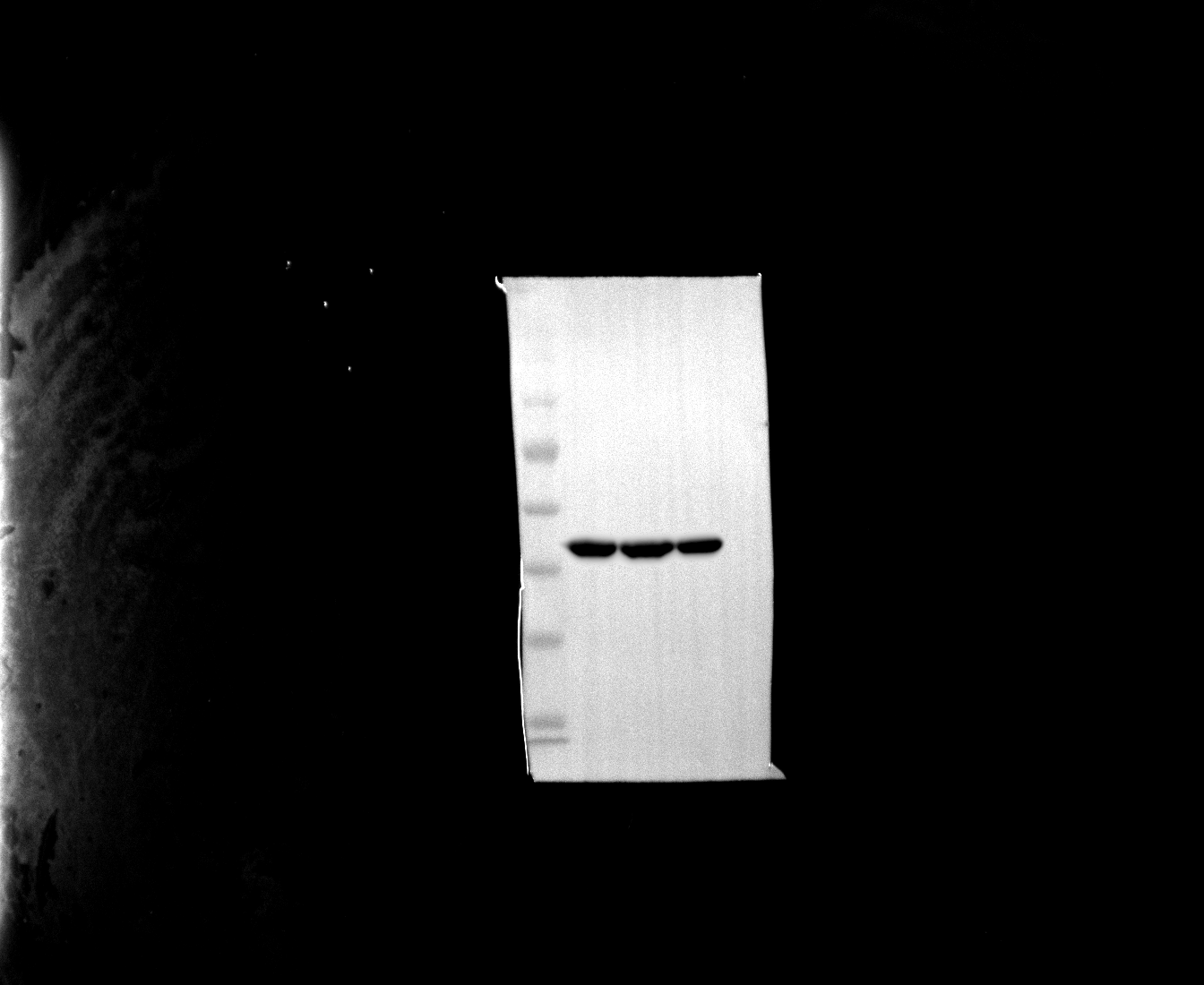


**DAPDH**


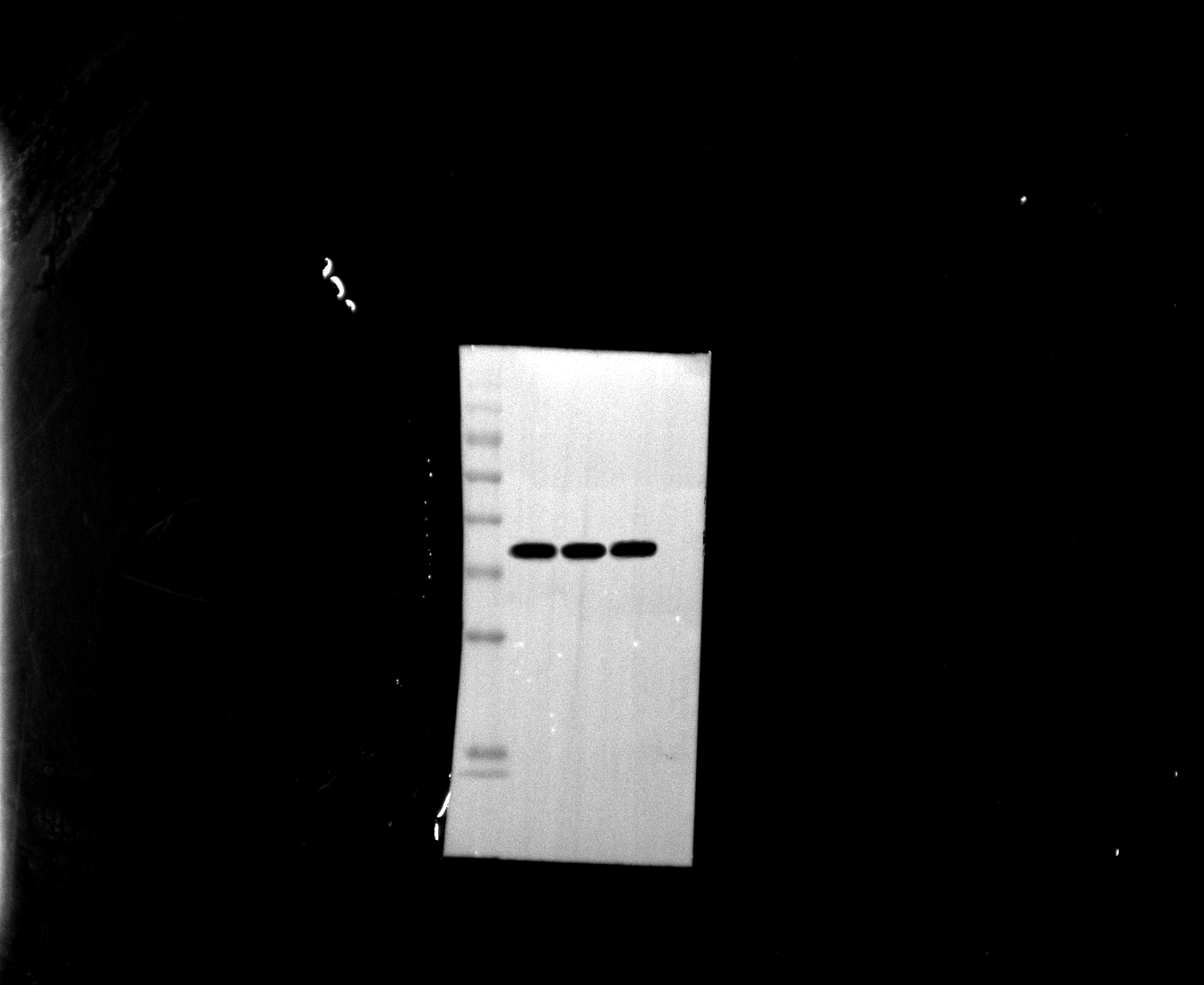

Supplement: S2 Raw images — (DOC) [file pone.0298353.s003.doc]
